# Divergent physiological strategies distinguish tolerant and plastic genotypes in elite Australian rice lines under limited irrigation

**DOI:** 10.3389/fpls.2026.1760397

**Published:** 2026-02-13

**Authors:** Yvonne Fernando, Markus Kuhlmann, Mark A. Adams, Vito Butardo

**Affiliations:** 1 Department of Chemistry and Biotechnology, Swinburne University of Technology, Hawthorn, VIC, Australia; 2 Institute of Plant Genetics and Crop Plant Research (IPK), Gatersleben, Germany

**Keywords:** Oryza sativa, epicuticular wax, non-photochemical quenching, stomatal density, leaf surface hydrophobicity, grain milling quality, field capacity, composite multi-trait index

## Abstract

**Introduction:**

Water scarcity threatens global rice production, necessitating identification of genotypes with improved water use efficiency (WUE) whilst maintaining productivity. Previous drought studies typically imposed severe stress conditions that compromised yield and quality, creating a knowledge gap regarding rice responses to moderate water limitation during vegetative growth. Here we show that 18 temperate japonica and 2 indica rice genotypes employ two distinct water conservation strategies under controlled limited water conditions (60–65% field capacity): inherent physiological tolerance versus adaptive phenotypic plasticity.

**Methods:**

We evaluated rice varieties under ponded and limited water treatments, integrating stomatal traits, chlorophyll fluorescence parameters, leaf carbon isotope composition (δ^13^C), and surface properties quantified via scanning electron microscopy and ATR-FTIR spectroscopy.

**Results:**

Inherently tolerant genotypes maintained stable photosynthetic performance through constitutively lower stomatal conductance and enhanced cuticular wax deposition. Conversely, adaptive genotypes exhibited pronounced physiological plasticity under water limitation. Notably, LW treatment induced significant enlargement of leaf surface papillae positioned over stomatal complexes, suggesting a potential structural mechanism contributing to reduced transpirational water loss. This represents a previously under-recognised adaptation in smooth-leaf Australian germplasm lacking protective trichomes. Mixed-effects modelling confirmed that photochemical traits and water-use traits responded most strongly to treatment, while reproductive and yield-related measurements indicated no major penalty under limited water. Carbon isotope discrimination (δ^13^C) validated superior intrinsic WUE in top-performing varieties.

**Discussion/conclusion:**

These complementary strategies provide multiple pathways for breeding water-efficient rice adapted to Australian temperate production systems under moderate water limitation without substantial yield loss.

## 1 Introduction

Water scarcity is an increasingly urgent global issue, particularly in regions where agriculture is a major economic driver (Food and Agriculture Organization of the United Nations, 2019; Rosa et al., 2020). Although the cultivation of rice in Australian is relatively small compared to other cereals, it plays a significant role in the agricultural sector and contributes substantially to the economy (Bajwa and Chauhan, 2017; Australian Government Department of Agriculture, Fisheries and Forestry, 2019). While the Australian rice industry is amongst the most water-efficient globally (RGA, 2023), it nonetheless faces substantial challenges due to fluctuating water availability driven by variability in climate and in competing demands from other sectors. Water scarcity poses a serious threat to global food security because rice, a staple food for billions of people, depends heavily on water as a semi-aquatic grass (Haonan et al., 2023; Hoque et al., 2021; Humphreys et al., 2006).

Selecting genotypes for water use efficiency (WUE) can help mitigate the impact of water scarcity on rice cultivation (Haonan et al., 2023; Mallareddy et al., 2023). Understanding mechanisms that influence WUE, particularly factors related to water loss through leaves, is essential to breeding and selecting water-efficient rice germplasm (Bramley et al., 2013; Lang et al., 2024). Leaf water loss occurs through both stomatal and non-stomatal pathways, making traits such as stomatal conductance, stomatal density, and cuticular or epicuticular wax deposition critical for regulating water conservation (Pitaloka et al., 2022, Pitaloka et al., 2021).

Stomatal traits, including density, size, and arrangement, directly influence gas exchange and transpiration (Pitaloka et al., 2022). Recent research has demonstrated that rice varieties with reduced stomatal density and smaller stomatal size exhibit improved WUE and biomass production under various growing conditions (Pitaloka et al., 2022; Phunthong et al., 2024). Non-stomatal traits such as cuticular wax composition and deposition also contribute substantially to plant water conservation, particularly under water-limited conditions (Srinivasan et al., 2008; Haque et al., 1992). Epicuticular waxes form a hydrophobic barrier on the leaf surface that reduces non-stomatal water loss while protecting against environmental stresses (Islam et al., 2009; Qin et al., 2011).

Most studies on WUE in rice have primarily focused on inducing severe drought conditions (Kamoshita et al., 2015; Praba et al., 2009), often resulting in reduced yield and grain quality (Blakeney, 1979; He et al., 2022; Ward et al., 2019). More carefully controlled water limitation can still induce stress responses without causing severe drought effects which reflects optimal water management regime on Australian rice farms. The vegetative stage is particularly critical in the rice life cycle, typically requiring irrigated conditions to establish robust growth. However, there remains a significant gap in understanding how rice responds to moderate water limitation during this stage, especially regarding the integration of stomatal and non-stomatal adaptations and their combined effects on grain yield and quality. Assessing all physiological traits, yield and milling quality components under such conditions is therefore essential to determine whether improved WUE can be achieved without compromising productivity.

The current study addresses this gap by examining the physiological and biochemical responses of Australian commercial elite rice lines to limited water stress during the vegetative stage. By maintaining water at 60-65% field capacity rather than inducing severe drought, this study sought to identify rice varieties that display adaptive water conservation strategies without compromising productivity. The specific objectives were to identify stomatal and non-stomatal leaf traits associated with WUE in diverse Australian commercial rice varieties under limited water conditions; find varieties with superior water conservation traits; and determine whether supplying limited water during the vegetative stage affects key yield and grain quality components. This integrated approach provides valuable insights for pre-breeding efforts aimed at developing rice varieties that maintain productivity under water-limited conditions.

## 2 Materials and methods

### 2.1 Rice germplasm

Twenty-one rice lines obtained from the Department of Primary Industries (New South Wales, Australia), were evaluated. These included 18 Australian temperate *japonica* commercial rice lines, two selected *indica* rice varieties (Pokkali and Purple) that are used as commercial cultivars, and the positive control Moroberekan. Moroberekan is a West African hybrid *japonica* x *indica* cultivar (Inukai et al., 1996) known for drought- and rice blast-resistance (Grondin et al., 2018). The rice varieties represented different elite grain types including medium grain, long grain, short grain, fragrant, and arborio developed or used as parental lines by the Australian rice industry (**Supplementary Table 1**).

### 2.2 Soil field capacity determination

A soil dry-down curve was developed for the potting media (70% composted pine bark 0–5 mm, 30% coco peat, pH 6.35, EC = 650 ppm, with 3 g/L Osmocote Exact 3-4M [19-9-10 + 2MgO + TE, ICL Specialty Fertilizers], 2 g/L Osmocote Exact 5-6M [15-9-12 + 2MgO + TE]) (Vinarao et al., 2021) to determine the FC for the limited water experiment. Four soil pots (square-shaped, height 17 cm, width 7 cm) containing five rice plants each (10-week-old, different varieties) were saturated with water and left in the glasshouse (average temperature: 27°C, relative humidity: 50%) without additional watering. The weights of the pots and soil moisture content (MC) were measured (Wireless soil moisture sensor, Ciderhouse Tech, AU) daily, and the plants’ drought response characteristics were observed throughout the experiment. Using early drought response such as slight leaf folding, drooping and leaf rolling, the wilting point was identified in the curve, and the midpoint from the beginning to the wilting point was selected as the FC to conduct the limited water experiment.

### 2.3 Glasshouse experiment

Two trials of experiments were conducted under two water treatments: ponded water (PW) and limited water (LW), in a glasshouse under controlled conditions at Swinburne University of Technology, Wantirna campus, Australia, from August 2023 to April 2024 and, from September 2024 to April 2025. A 16 hours of daylight at 25-30°C, 8 hours of night-time at 15-20°C, 50% average relative humidity, and light intensity of 30,000 - 35,000 lux were maintained in the glasshouse, according to optimal conditions of the rice growing season in the Riverina region in Australia.

All seeds were primed at 40°C for four days and dehulled before planting. Pots (square-shaped, height 17 cm, width 7 cm) were constructed with meshed bases, enabling roots to extend beyond the pot volume into surrounding ponded trays, thereby minimising artificial root confinement. Pots were spaced 40 cm apart to prevent canopy interference. Soil moisture was continuously monitored using wireless soil moisture sensors, with daily readings used to adjust irrigation to maintain required field capacity, as determined from a pre-established soil moisture calibration curve. Pots with the same weight (325g) were prepared and placed in the water ponds in the glasshouse two days before seed planting to settle the potting mix. Four seeds were planted in each pot, and thinned at the three-leaf stage, leaving one plant per pot. In each trial, eight pots were prepared for each rice genotype, with four replicates conducted per variety under each water condition. The experiment was conducted using a complete randomized block design (CRBD).

When the plants in the LW plants pond reached the three- to five-leaf stage, water was removed from the LW plants. The MC and FC determined through the dry-down curve were maintained throughout the entire vegetative growth stage. Each pot was re-irrigated individually upon reaching the booting stage in a separate pond, while maintaining the original CRBD.

### 2.4 Stomatal conductance (g_s_).

Stomatal conductance (g_s_) measures the rate at which CO_2_ enters and water vapour exits the leaf through stomata. Stomata are small pores on the leaf surface that regulate gas exchange, allowing CO_2_ to enter for photosynthesis and water vapor to exit through transpiration. Higher g_s_ allows more CO_2_ for photosynthesis but also increases water loss, while lower g_s_ helps conserve water under drought or limited water conditions (Bertolino et al., 2019; Asargew et al., 2024). An SC-1 Leaf porometer equipped with desiccants in the sensor head (Decagon Devices Inc., USA) was used to measure g_s_ (Matsuo et al., 2010; Ramos-Fernández et al., 2024; Wu et al., 2020; Manavalan et al., 2011). Abaxial g_s_ was measured on the middle, widest region of the fully expanded second leaf of the primary tiller for each individually grown plant, ensuring consistent positioning across samples.

### 2.5 Stomatal density

Stomatal density (SD) is the number of stomata per unit leaf area. Higher SD can increase CO_2_ uptake but may also increase water loss, whereas lower SD helps conserve water under drought or limited water conditions (Bertolino et al., 2019). Imprints of the vegetative leaf epidermis were taken from the middle wider part of the fully expanded second mature leaves (Cowling et al., 2021; Pathoumthong et al., 2022; Wu and Zhao, 2017). A thin layer of fast-drying clear nail polish (Rimmel, London) was applied and air-dried for about 10 minutes. Dry nail polish was peeled off using clear tape and placed on a microscope slide. Imprints of the abaxial leaf surfaces were taken, and stomata were observed in a 600 μm × 450 μm region area through an EVOS 5000 Microscope (Thermo Fisher Scientific) at 200× magnification and converted into the number of stomata/mm².

### 2.6 Cuticular and epicuticular wax and flavonols

Cuticular and epicuticular wax (CEW) forms a protective layer on the leaf surface that reduces water loss and shields the plant from environmental stresses. Flavonols are specialised plant metabolites that act as antioxidants, protecting leaves from oxidative damage, UV radiation, and other stressors (Bernaola et al., 2021; Haque et al., 1992; Laoué et al., 2022). Attenuated Total Reflectance - Fourier Transform Infrared (ATR-FTIR) analysis (Nicolet iS5 Spectrometer, Thermo Fisher Scientific, Waltham, MA, USA) was used to analyse and semi-quantify the CEW and flavonols on fresh rice leaves (Sharma and Uttam, 2016; Willick et al., 2018; Yun et al., 2024). Spectra were measured in the 400 to 4000 cm^-^¹ range using OMNIC software, with each spectrum representing the average of 32 scans at a resolution of 4 cm^-^¹. The spectral region 2800–3000 cm^-^¹ was used to detect aliphatic components of the leaf cuticle, including cutin, epicuticular waxes, and cutan (Heredia-Guerrero et al., 2014). The fresh leaves were treated according to the methods described by Nguyen et al. (2018) and then the leaves were sputter-coated with gold, and the papillae structure was then observed using a scanning electron microscope (SEM) to compare changes between PW and LW leaves (Xiang et al., 2017).

Papillae are dense, microscopic outgrowths of the leaf epidermis found in many plant species, including rice, where they contribute to water retention, pathogen resistance, and structural support (Pitaloka et al., 2021; Yoo et al., 2011). Papillae were manually counted within a defined region of interest corresponding to a known surface area (µm²) using the SEM images and then converted into mm^2^. To measure the area of each papilla apex (top surface), ImageJ (v1.54), a software tool widely used for leaf surface and morphological measurements (Wuyts et al., 2010; Chatterjee et al., 2016), was used by outlining each apex with the selection tool, after which the calibrated “Measure” function was applied to obtain papilla apex area (µm²).

The rate of water loss from detached leaves provides an indirect measure of cuticular and stomatal water retention capacity, reflecting the leaf’s ability to conserve water under limited availability (Nguyen et al., 2018; Zhang et al., 2025a; Zhou et al., 2015). The rate of water loss was measured using detached leaves at week 10. Plants were kept in the dark for 4 hours to ensure full stomatal closure. Leaves were weighed under low-intensity light conditions, with the cut edge sealed using petroleum jelly (Vaseline; Unilever, USA) to minimize water loss from the excised surface. Leaf weight was recorded using a three-decimal analytical balance (Ohaus, USA) over a total period of 1.5 hours. Percentage water loss at each time point was calculated by dividing the leaf weight at that time by the initial weight and expressing it as a percentage.

### 2.7 Photosynthetic parameters

The MultispeQ V2.0 device (PhotosynQ Inc., USA) was used to measure leaf photosynthetic parameters (Zuo et al., 2024; Oo et al., 2023). These included relative chlorophyll content (RCh), leaf temperature (LT), and chlorophyll fluorescence parameters such as the effective quantum yield of photosystem II (ΦPSII), the maximum quantum efficiency of PSII (Fv/Fm), total non-photochemical quenching (ΦNPQt), and the fraction of light energy dissipated through non-photochemical quenching (ΦNPQ).

### 2.8 Leaf surface hydrophobicity

Measuring contact angles provides insights into leaf surface properties, water retention capacity, and plant adaptation mechanisms under contrasting water regimes. Contact angles of the leaf surfaces were measured using the FTA 1000 Drop Shape Analyzer (First Ten Angstroms, Inc., USA) to assess surface water repellency and microstructural adaptations (Kwon et al., 2014; Zhu et al., 2014) under limited water conditions. Higher angles indicate a more hydrophobic (water-repellent) surface and lower angles indicate a more hydrophilic (wettable) surface providing an indirect assessment of cuticular wax content, composition, and surface microstructure (Zhu et al., 2014).

### 2.9 Carbon isotope composition

Leaf carbon isotope composition (δ¹³C) was measured because it provides an established integrative proxy for intrinsic water-use efficiency (iWUE) in C_3_ species. iWUE is defined as the ratio of CO_2_ assimilation rate to stomatal conductance, and δ¹³C reflects long-term variation in this balance through carbon isotope discrimination during photosynthesis (Farquhar et al., 1989; Impa et al., 2005; Jiang et al., 2024). Healthy, fully expanded second leaves from each genotype were collected at week 10. Leaf samples were oven-dried at 65°C for 72 h, then finely ground using a ball mill. Approximately 2 mg of homogenised material was weighed into tin capsules (Sercon, UK) and sealed. Capsules were combusted in an elemental analyser (CE-Instruments EA 1110, UK), and the resulting CO_2_ and N_2_ gases were transferred to an isotope ratio mass spectrometer (IRMS; Micromass IsoPrime, UK) for isotopic analysis. Carbon isotope composition (δ¹³C, %; values reported relative to the VPDB standard), total carbon (C %), and total nitrogen (N %) were quantified using the EA–IRMS system following standard procedures (Cernusak et al., 2004).

### 2.10 Yield and reproductive performance measurements

To evaluate whether moderate water limitation affected reproductive performance and to ensure relevance to grain productivity, key yield-related traits were quantified at physiological maturity. Panicle number per plant was recorded by manually counting all fully developed panicles. Filled and unfilled grains were separated by visual inspection and gentle pressure, and only fully developed seeds were retained for subsequent analyses. Filled grains were oven-dried at 30°C until seed moisture equilibrated to approximately 12–14% (IRRI), consistent with postharvest drying standards, to preserve viability for subsequent germination. Grain weight per plant was then determined as the dry mass of these viable filled grains. Filled grain percentage (%) was calculated as:


$$\text{Filled Grain }\%=\frac{\text{Number of Filled Grains}}{\text{Total spikelet number}}\times 100$$

Thousand grain weight (TGW) was estimated from the dry mass of 1,000 randomly selected filled grains. Above-ground shoot biomass was dried to constant mass prior to weighing, and harvest index (HI) was calculated as:


$$\text{HI}=\frac{\text{Filled Grain Dry Weight}}{\text{Total Aboveground Shoot Dry Biomass}}\;\;$$

### 2.11 Grain milling quality

Paddy rice samples from each genotype and water treatment were cleaned, equilibrated, and subsequently processed to assess milling quality. Ten grams (10g) of paddy grains were first dehulled using a laboratory rice husker (TR-260 Automatic Rice Husker, Kett, Japan) to obtain brown rice. Brown rice yield (BRY) was calculated as the percentage ratio of brown rice weight to the initial paddy weight. Brown rice samples were then polished using a laboratory rice polisher (PEARLEST TP-3000 Grain Polisher, Kett, Japan). Polishing was performed for 60 s at a constant load, after which milled rice yield (MRY) was determined as the percentage ratio of milled rice weight to the initial paddy weight. Following polishing, milled rice samples were visually inspected and separated into whole and broken grains. Head rice recovery (HRR) was expressed as the percentage of intact whole kernels (≥75% of full grain length) relative to the initial paddy weight.

### 2.12 Statistical analysis

All analyses were conducted using R (v4.5.1; RStudio v2025.09.2), Python (v3.13.7, Jupyter Notebook), and GraphPad Prism (v10.6.0). Two statistical frameworks were applied according to data structure. For traits measured within a single experiment with balanced replication (e.g., stomatal traits, papillae number and area, milling quality parameters), treatment and subspecies effects were analysed using two-way ANOVA, followed by *post-hoc* comparisons where appropriate.

For traits measured across multiple trials or involving hierarchical data structure (e.g., PhotosynQ fluorescence parameters, δ¹³C and leaf C–N traits, whole-plant integrative responses), linear mixed-effects models were fitted using lme4 (Bates et al., 2015), with treatment modelled as a fixed effect and genotype and/or trial included as random effects. This approach enabled robust assessment of treatment, trial, and treatment × trial interaction effects, appropriately accounting for repeated measurements and non-independence of observations, and is widely applied in rice and plant physiological research (Johnson et al., 2024; Zhang et al., 2025b). Estimated marginal means were derived using emmeans (Lenth, 2023). Correlation analysis, PCA, clustering, and genotype ranking were performed in Python. Papillae apex area was quantified using ImageJ (v1.54). Statistical significance was accepted at p< 0.05.

## 3 Results

The Results section begins by outlining soil MC and FC determinations used to establish the LW experiment. It then presents the effects of water limitation on key leaf physiological traits, including stomatal conductance, stomatal density, wax composition, photosynthetic performance, and leaf surface hydrophobicity. Genotype responses are summarised through ranking and the Composite Multi-Trait Index (CMTI), followed by multivariate (PCA, correlations) and mixed-effects model analyses to quantify treatment, trial, and interaction effects. Finally, δ¹³C and leaf C-N traits are examined to assess whole plant water use strategies under PW and LW regimes.

### 3.1 Soil dry-down curve

Leaf folding, drooping became evident in most plants on Day 7, as shown in **Supplementary Figure 1**. Therefore, the moisture content on Day 4 (25-28%) were used as a guide to maintaining LW while avoiding drought response throughout the vegetative stage of the LW plants. At that MC, FC of the potting mix was ~60-65% of the original FC.

### 3.2 Stomatal conductance (g_s_)

**Figure 1:**
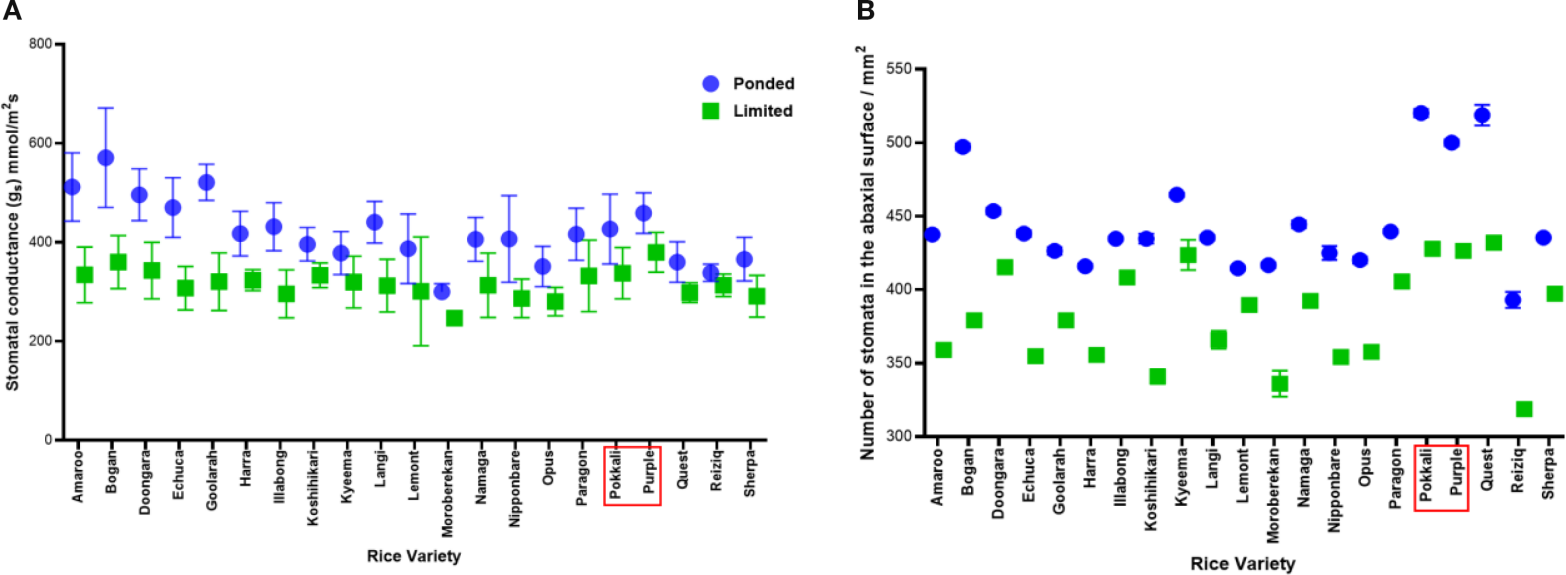
Stomatal traits of rice varieties under PW and LW conditions at week 10. **(A)** g_s_ was reduced under LW compared with ponded conditions (PW) (two-way ANOVA, Trt p< 0.001). *Indica* varieties (red square) had higher gs than *japonica* across treatments (p = 0.036) **(B)** Stomatal density on the abaxial leaf surface decreased under LW (two-way ANOVA, Trt p< 0.001). *Indica* varieties maintained higher SD than *japonica* across treatments (p< 0.001). Data represent mean ± SEM, n = 21 varieties with 8 biological replicates per variety per treatment and 12 technical replicates.

All rice varieties exhibited reduced gs under LW compared with PW (two-way ANOVA, p< 0.001), indicating that water limitation restricted stomatal opening and thus CO_2_ uptake (**Figure 1A**). Subspecies also differed significantly, with *indica* averaging higher g_s_ than *japonica* varieties across both water treatments (p = 0.036), while the interaction between treatment and subspecies was not significant (p = 0.43), indicating a consistent water response across subspecies. *Post-hoc* comparisons under PW showed that Bogan had the highest g_s_ (“a”), whereas Moroberekan had the lowest (“e”), with most other varieties showing intermediate values (**Supplementary Figure 2**). Reduced g_s_ in LW plants is likely to limit CO_2_ assimilation, potentially reducing photosynthetic efficiency. Mean gs values by treatment × subspecies were: LW *indica* 355 ± 50 mmol m^-^² s^-^¹< LW *japonica* 312 ± 53 mmol m^-^² s^-^¹< PW *indica* 442 ± 59 mmol m^-^² s^-^¹< PW *japonica* 422 ± 86 mmol m^-^² s^-^¹.

### 3.3 Stomatal density

All rice varieties exhibited reduced SD on the abaxial leaf surface under LW compared with PW conditions (two-way ANOVA, p< 0.001), likely representing an adaptive response to conserve water by limiting transpiration (**Figure 1B**). Subspecies also differed significantly, with *indica* averaging higher SD than *japonica* species across both water treatments (p< 0.001), suggesting greater potential for CO_2_ uptake but also a higher potential for water loss. The interaction between treatment and subspecies was not significant (p = 0.10), indicating that both subspecies respond similarly to water limitation by reducing SD. *Post-hoc* comparisons among varieties under PW showed that Pokkali and Quest had the highest SD (“a”), whereas Moroberekan and Reiziq had the lowest (“h–i”), with most other varieties showing intermediate values (**Supplementary Figure 3**). Variety-specific differences highlight genotypic variation in potential gas exchange and water-use strategies, with clear genotype × treatment patterns evident when comparing varieties within PW and LW conditions. Mean SD values by treatment × subspecies were: PW *indica* 510 ± 12 mm^-^² > PW *japonica* 439 ± 29 mm^-^² > LW *indica* 427 ± 7 mm^-^² > LW *japonica* 377 ± 32 mm^-^² (**Figure 1**).

### 3.4 Cuticular and epicuticular wax and flavonols

In the ATR-FTIR spectra of fresh rice leaves (**Supplementary Figure 4**), peaks associated with CEW were observed in the 2800–3000 cm^-^¹ region, corresponding to symmetric and asymmetric C–H stretching vibrations of methyl and methylene groups, which arise from aliphatic components of the leaf cuticle (cutin, cuticular waxes, and cutan), as biochemically validated by earlier studies (Phansak et al., 2021; Sharma and Uttam, 2016; Heredia-Guerrero et al., 2014; Tran et al., 2025). Peaks associated with flavonols were detected mainly in the fingerprint region, including 1125–1140 cm^-^¹ (C–H bending of the aromatic ring), 1205–1225 cm^-^¹ (aromatic C=C stretching), 1270–1310 cm^-^¹ (C=C stretching and O–H bending), 1435–1475 cm^-^¹ (aromatic ring stretching and O–H bending), and 1605–1620 cm^-^¹ (C=O and C_2_=C_3_ stretching), which have been previously validated through biochemical analyses and FTIR assignments (Herath et al., 2024; Krysa et al., 2022).

**Figure 2:**
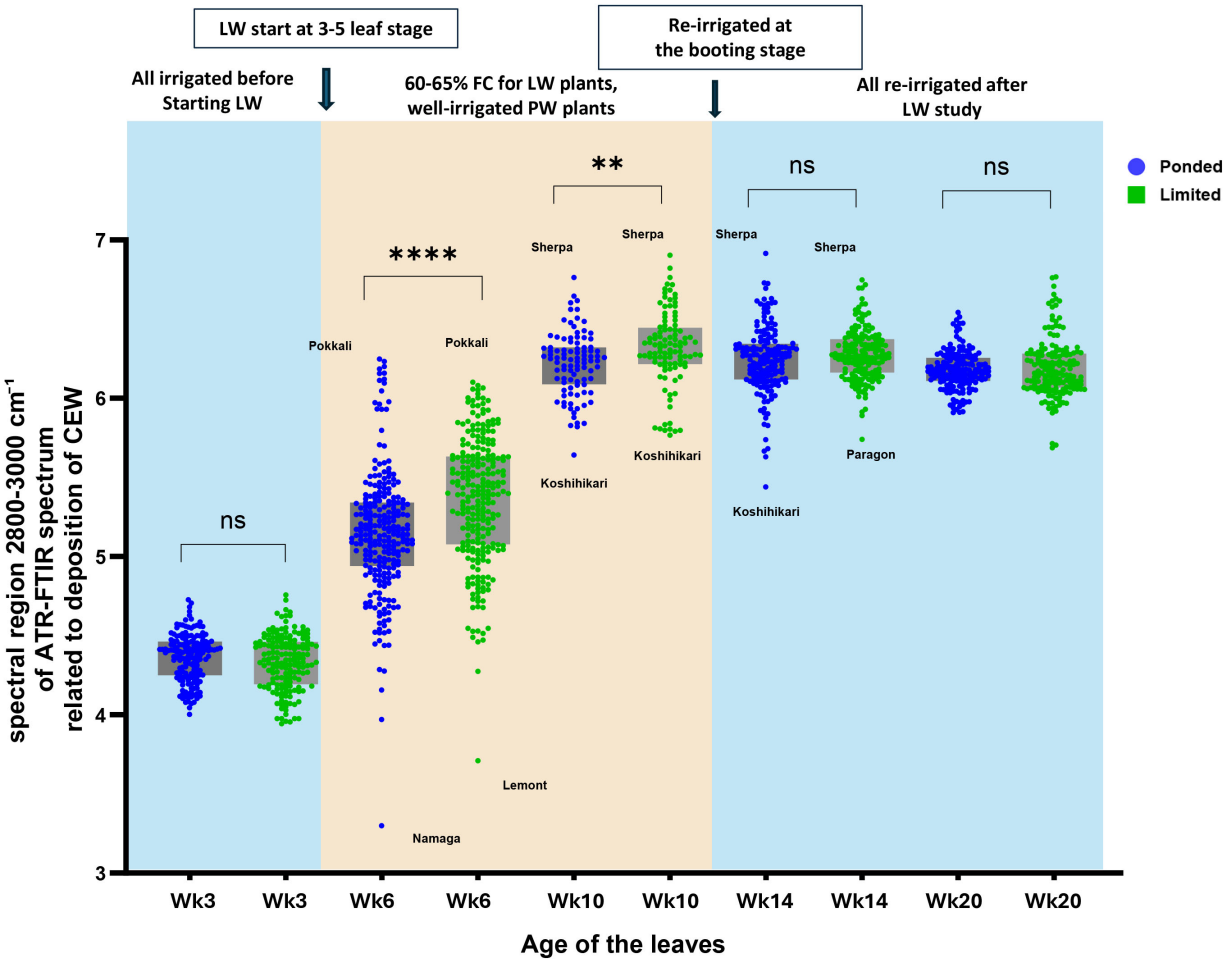
Time-course of CEW deposition on rice leaf surfaces under PW and LW conditions. The brown shaded area indicates the experimental period when LW treatment was applied. LW leaves showed significantly higher CEW deposition during water stress at weeks 6 and 10, with differences diminishing after re-irrigation. Data represent mean ± SEM, n = 21 varieties with 8 biological replicates per variety per treatment and 12 technical replicates. Statistical significance is indicated as ** p < 0.01 and **** p < 0.0001; ns, not significant.

**Figure 3:**
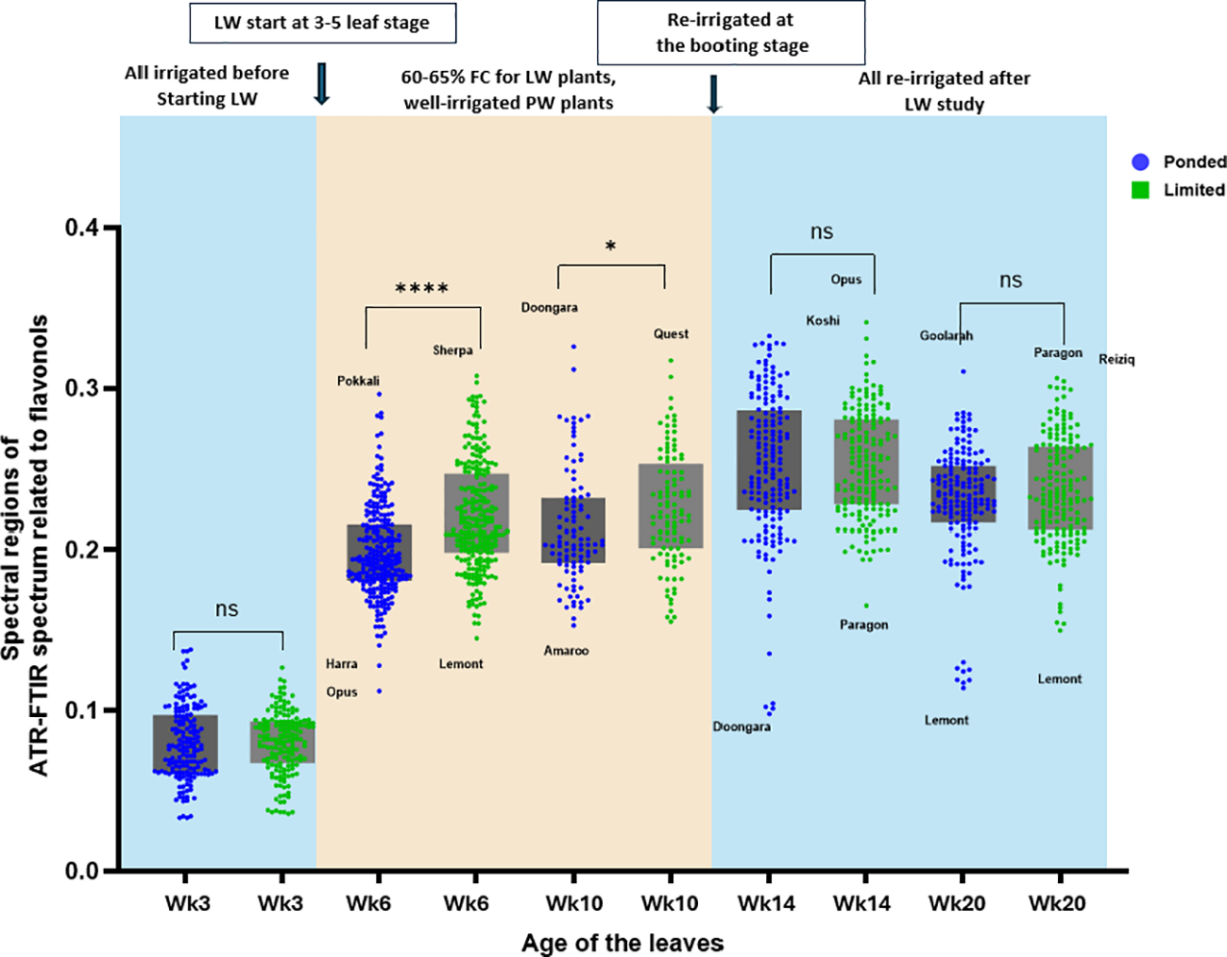
Time-course of flavonol content on rice leaf surfaces under PW and LW conditions. The brownshaded area indicates the experimental period when LW treatment was applied. LW leaves exhibited significantly higher flavonol content during water stress, with differences persisting after re-irrigation. Data represent mean ± SEM, n = 21 varieties with 8 biological replicates per variety per treatment and 12 technical replicates. Statistical significance is indicated as * p < 0.05, **** p < 0.0001; ns, not significant.

**Figures 2**, **3** show a time series analysis of CEW deposition and flavonol formation on the leaves derived from the ATR-FTIR spectrum. At Week 3 (Wk3), all plants were under PW conditions just before starting the LW experiment, resulting in no significant difference between the PW and LW readings. During the water stress period, at weeks 6 (Wk6) and 10 (Wk10), the leaves of LW grown plants show higher spectral signals for CEW and flavonols, and they achieved the optimum CEW deposition at an increasing rate. The salt tolerant cultivar Pokkali (Tiwari et al., 2023) and stress resistant cultivar Sherpa (Vinarao et al., 2023) produced a higher amount of CEW and flavonols during the water stress. After re-irrigation, no significant differences were detected between PW and LW for either CEW or flavonols at Wk14 or Wk20. However, flavonol intensities declined from Wk14 to Wk20 in both treatments, indicating a gradual biochemical recovery and reduced stress-related secondary metabolite production once an adequate water supply was restored.

**Figure 4:**
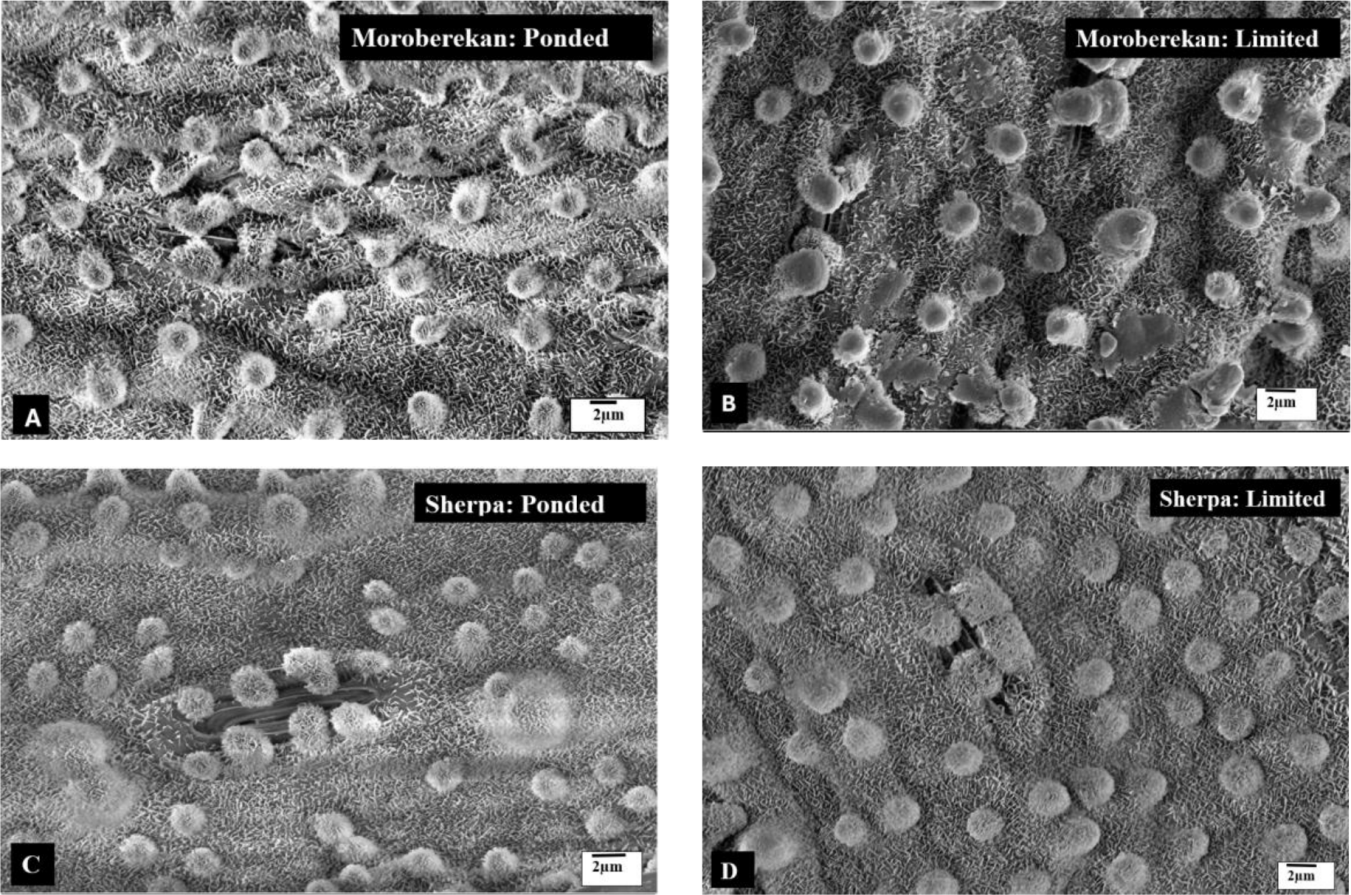
SEM images showing epicuticular wax structures on leaf surfaces of *Oryza sativa* cultivar Moroberekan and cultivar Sherpa. **(A, C)** PW leaf surface showing normal epicuticular wax crystals and papillae. **(B, D)** LW leaf surface displaying larger and more elevated papillae that cover stomatal openings. Scale bars represent 2 µm. Images shown are representative of 4 biological replicates per treatment with 3 technical replicates each.

The SEM images showed that both the LW and PW leaves had the same crystal-like epicuticular wax structures, but the LW leaves exhibited larger and more elevated papillae on their surface. It was observed that unlike the papillae on the PW leaves, the larger papillae on the LW leaves cover the stomata openings well (**Figure 4**), suggesting a structural change that may help restrict water loss under limited water conditions.

Papillae on the adaxial leaf surface differed among rice varieties in both number and size. Papillae number varied significantly among varieties (two-way ANOVA, p< 0.001), with a marginal effect of water treatment (p = 0.064) and no significant variety × treatment interaction (p > 0.9), indicating similar responses under PW and LW conditions. *Indica* varieties had higher papillae numbers than *japonica* (p< 0.001), regardless of water regime. This consistent difference between subspecies indicates inherent variation in surface wax structure abundance. Under PW conditions, *indica* varieties generally had the highest numbers, whereas Moroberekan and Namaga (*japonica*) had the lowest (**Supplementary Figure 5**). Mean papillae numbers by treatment × subspecies were: LW *indica* 37,931 ± 3,047 > PW Indica 37,568 ± 3,874 > LW Japonica 29,743 ± 4,076 > PW Japonica 29,240 ± 4,103. Given that Moroberekan displayed the lowest papillae number but higher CEW content in the ATR-FTIR measurements, papilla apex surface area was quantified from SEM images using ImageJ. Papillae apex area also differed significantly among varieties (two-way ANOVA, p< 0.001), with strong effects of water treatment (p< 0.001) and a significant variety × treatment interaction (p< 0.001), indicating variety-specific responses. Moroberekan had the largest papillae area under PW. Subspecies had no significant effect (p = 0.790) or interaction with treatment (p = 0.596). Under LW, papillae area increased across most varieties, with Moroberekan and Sherpa showing the largest values (**Supplementary Figure 6**). This enlargement under LW suggests that papillae expansion, rather than increased papillae numbers, was the dominant structural adjustment to water limitation. Mean papillae areas by treatment × subspecies were: LW *indica* 4.80 ± 0.26 μm² > LW *japonica* 4.78 ± 0.56 μm² > PW *japonica* 3.97 ± 0.49 μm² > PW *indica* 3.91 ± 0.58 μm², showing that water limitation increased papillae size similarly in both subspecies.

### 3.5 Leaf water loss

All LW leaves exhibited lower water loss (%) compared with their PW counterparts (**Supplementary Figure 7**). Moroberekan and Sherpa showed the lowest water loss under both PW and LW conditions, indicating enhanced barrier properties of their leaf surfaces against water loss. In contrast, Kyeema exhibited the highest water loss in both treatments. These results are consistent with previous observations in this study, where Moroberekan and Sherpa displayed higher CEW content on their leaf surfaces, supporting a protective role of epicuticular wax in limiting water loss.

### 3.6 Photosynthetic parameters

**Figure 5:**
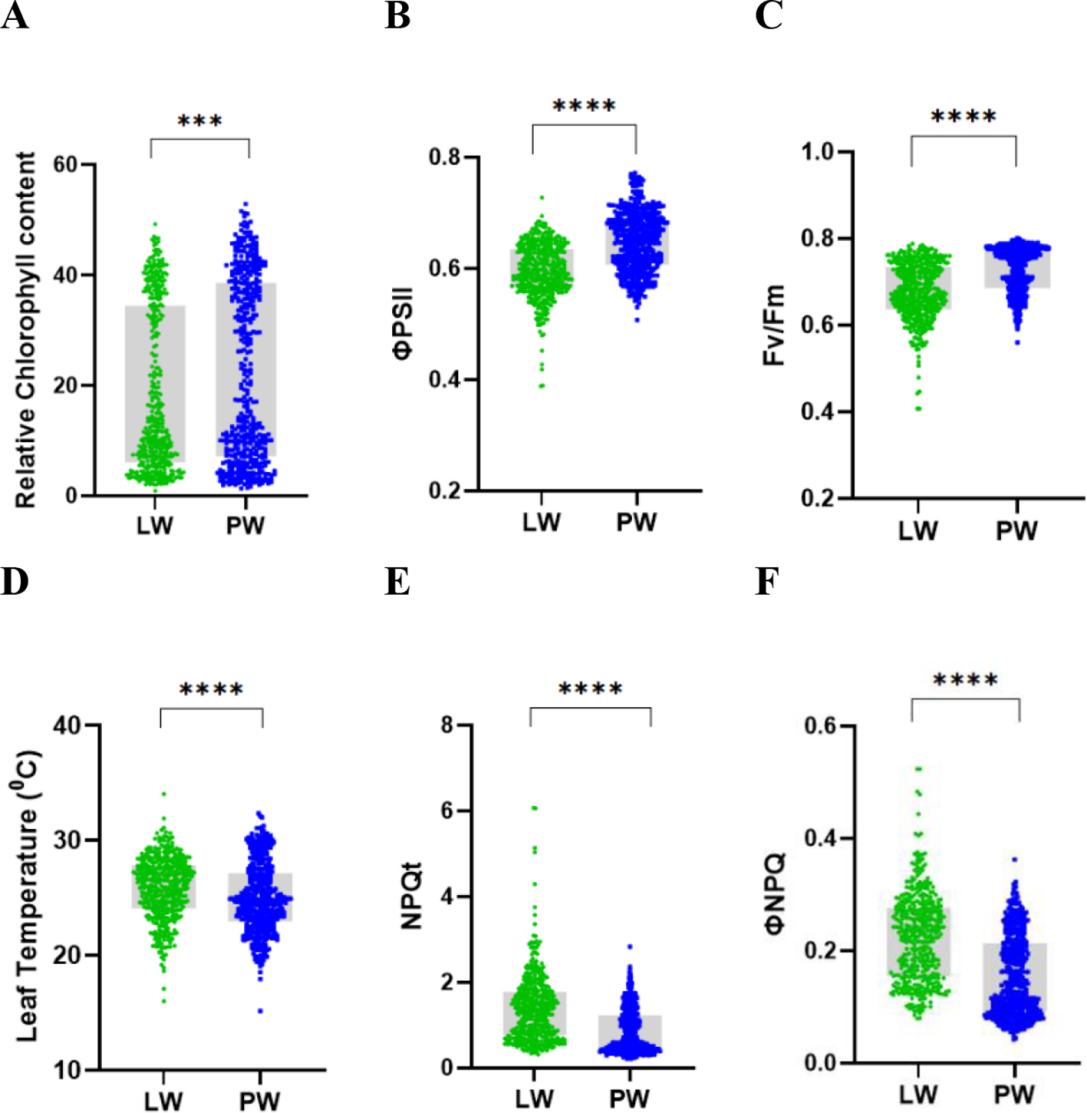
Photosynthetic parameters of rice varieties under PW and LW conditions at week 10. **(A)** Relative chlorophyll content, **(B)** ΦPSII, **(C)** Fv/Fm, **(D)** leaf temperature, **(E)** NPQt and, **(F)** ΦNPQ. LW plants showed significant decreases in photosynthetic efficiency parameters and increases in photoprotective mechanisms. Data represent mean ± SEM, n = 21 varieties with 8 biological replicates per variety per treatment and 12 technical replicates. Statistical significance is indicated as *** p < 0.001 and **** p < 0.0001.

LW plants showed reduced RCh, ΦPSII, and Fv/Fm compared with PW plants, indicating lower photosynthetic efficiency due to limitations in CO_2_ assimilation through stomata (**Figures 5A–C**). Higher leaf temperatures were observed in LW plants, likely resulting from reduced transpiration (**Figure 5D**). Both total non-photochemical quenching (ΦNPQt) and regulated non-photochemical quenching (ΦNPQ) were higher in LW plants (**Figures 5E, F**). While ΦNPQ represents the fraction of absorbed light energy dissipated as protective heat, ΦNPQt reflects all non-photochemical energy dissipation processes, including both regulated and unregulated mechanisms. The elevated ΦNPQt and ΦNPQ in LW plants indicate an increased need to safely dissipate excess light energy under water-limited conditions due to their reduced photosynthetic capacity.

### 3.7 Leaf surface hydrophobicity

Leaf contact angles were measured at Wk10 (**Supplementary Figure 8**) showed clear differences between water treatments. LW leaves exhibited higher contact angles than PW leaves, indicating increased leaf surface hydrophobicity under LW conditions. A higher contact angle reflects a more water-repellent surface, which is advantageous in dry conditions because it reduces cuticular water loss and limits leaf wetting. Varietal differences were also evident. Sherpa displayed the highest contact angle, consistent with its stronger CEW signal in ATR-FTIR measurements. This likely reflects an increase in epicuticular wax deposition and associated surface microstructure, contributing to a more hydrophobic leaf surface under LW conditions. The observed increase in contact angle under LW indicates that rice varieties can adjust leaf surface wettability in response to water limitation (Zhu et al., 2014).

### 3.8 Top-performing genotypes per trait

A summary of the most frequently ranked top-performing genotypes is presented in **Table 1**. This table consolidates genotypes that repeatedly appeared among the top five performers across traits and treatments, highlighting consistent physiological and structural advantages relevant to WUE and drought resilience. Moroberekan, Pokkali, Sherpa, Doongara, and Bogan are among the genotypes that feature most frequently, indicating they combine several favourable traits, photochemical, anatomical, or stomatal, across environments. Other varieties (such as Paragon, Nipponbare, Amaroo, and Goolarah) excel in a more limited subset of traits. This summary provides an integrated view of multi-trait performance, helping identify parental genotypes with consistent physiological and structural advantages that warrant priority in future WUE-focused evaluation and breeding program for the Australian and other temperate rice industry.

**Table 1:** Summary of top-performing genotypes across photochemical, anatomical, and stomatal traits in two trials under PW and LW conditions.

Genotype	Traits appearing in top 5	Key strengths	WUE implication
Moroberekan	ΦPSII, Fv/Fm, RCh, CEW, SD, g_s_	High photosynthetic efficiency and structural resilience	Strong WUE potential via photosystem and structural traits
Pokkali	ΦPSII, Fv/Fm, ΦNPQ, CEW, SD, g_s_, NPQt	Efficient photoprotection, cuticle and stomatal regulation	Likely efficient photoprotection and moderate WUE
Doongara	ΦPSII, Fv/Fm, RCh, CEW, g_s_	Balanced photosystem and structural traits	Moderate WUE through structural and photosystem traits
Sherpa	ΦPSII, Fv/Fm, ΦNPQ, CEW, g_s_	Stable photochemistry and moderate photoprotection	Consistent WUE under variable conditions
Paragon	Fv/Fm, RCh, LT, SD	Photosystem II stability and thermal/light regulation	Moderate to high WUE via photochemical and thermal traits
Amaroo	ΦNPQ, Fv/Fm, LT, CEW	Efficient light capture and moderate photoprotection	Moderate WUE with balanced photochemistry
Goolarah	ΦNPQ, LT, CEW	Light energy dissipation, moderate structural traits	Moderate WUE
Bogan	ΦPSII, Fv/Fm, RCh, g_s_, SD	Photosystem stability and structural robustness	Consistent WUE under stress
Nipponbare	ΦPSII, Fv/Fm, LT, ΦNPQ	Photosystem efficiency and moderate photoprotection	Moderate WUE
Illabong	ΦNPQ, Fv/Fm	Balanced photochemistry and energy dissipation	Moderate WUE

ΦPSII, effective quantum yield of photosystem II; Fv/Fm, maximum quantum efficiency of PSII; RCh, relative chlorophyll content; CEW, cuticular and epicuticular wax; SD, stomatal density; g_s_, stomatal conductance; ΦNPQ, regulated non-photochemical quenching; ΦNPQt, total non-photochemical quenching; LT, leaf temperature.

### 3.9 Principal component analysis

**Figure 6:**
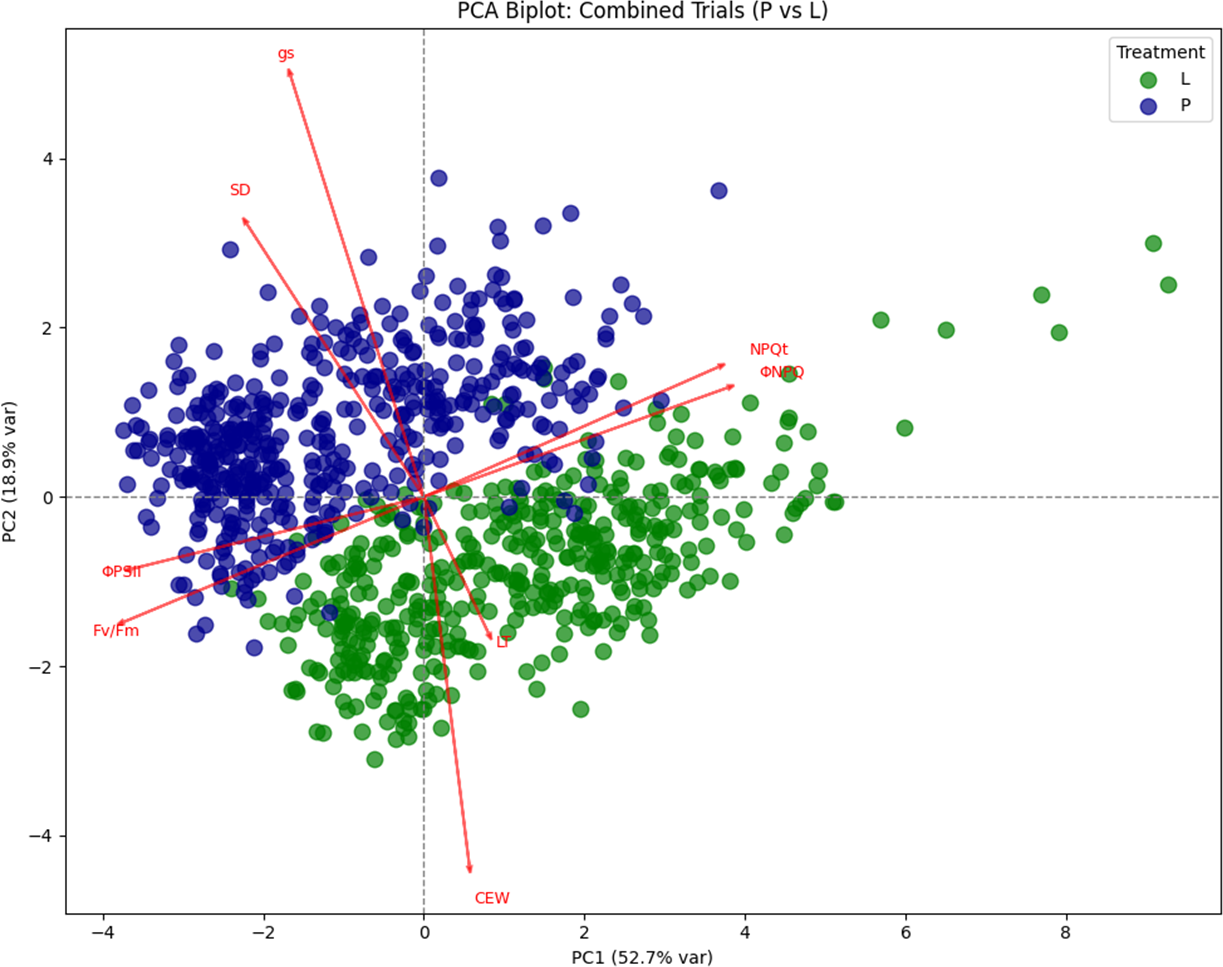
PCA biplot of physiological and structural traits under PW and LW conditions. Arrows show trait loadings, highlighting treatment- and trial-specific patterns in photosynthesis, stomatal traits, and structural adaptations.

As shown in **Figure 6**, g_s_ (SC), stomatal conductance (SD), ΦPSII, and Fv/Fm vectors point toward PW conditions, indicating higher stomatal activity and photosynthetic efficiency. In contrast, NPQt, ΦNPQ, LT and CEW vectors align with LW, reflecting structural and photoprotective adaptations to water limitation. Overall, the PCA separates genotypes based on photosynthetic, structural, and water-use traits, highlighting distinct strategies under PW versus LW conditions. The first principal component (PC1) had an eigenvalue of 4.22 and explained 52.7% of the total variance, while the second principal component (PC2) had an eigenvalue of 1.52 and accounted for an additional 18.9% of the variance. Together, PC1 and PC2 captured approximately 71.6% of the total variance, indicating that these two components effectively summarize the majority of variation in the dataset. This supports the use of a two-dimensional PCA plot to effectively visualize trait relationships and genotype differentiation.

### 3.10 Correlation structure

Correlation matrices revealed biologically coherent relationships among rice physiological traits under LW and PW treatments (**Supplementary Figure 9**). Strong correlations (r ≥ 0.5) were largely observed among chlorophyll fluorescence parameters, Fv/Fm, ΦPSII, ΦNPQ and NPQt reflecting close functional interdependence of photosystem II efficiency and photoprotective energy dissipation. Negative associations, such as between Fv/Fm and ΦNPQ, highlight typical energy trade-offs under water stress, where reduced photosynthetic efficiency coincides with increased thermal energy dissipation. Non-photosynthetic traits, including CEW and g_s_, showed weaker or inconsistent correlations with fluorescence traits, consistent with differences in their temporal regulation, where wax and structural traits reflect longer-term developmental investment while g_s_ is highly dynamic. The weaker correlations therefore more likely reflect inherent trait behaviour rather than measurement variability. Together, the correlation structure supports coordinated regulation of photochemistry under water limitation while indicating that structural and surface traits contribute independently to water-use strategies under PW and LW conditions.

### 3.11 Effect sizes of traits under contrasting water treatments

To quantify the magnitude of treatment effects on rice traits and understand genotype sensitivity to environmental variation, Cohen’s d was calculated (**Supplementary Figure 10**) for each trait under PW versus LW conditions. Large effect sizes for g_s_, SD, leaf CEW, ΦPSII, ΦNPQ, Fv/Fm, and NPQt indicate that water-use regulation and key photochemical processes are highly responsive to water limitation, reflecting adaptive physiological and structural adjustments to maintain photosynthetic performance under stress. In contrast, small effects for LT and RCh indicate that structural or slower-response traits are relatively insensitive to short-term water stress.

To identify the traits most strongly associated with iWUE Partial Least Squares (PLS) regression was performed using δ¹³C as the response variable. VIP scores revealed that gs, leaf CEW, and SD, together with ΦPSII, were the strongest contributors to δ¹³C variation (VIP > 1), indicating their value as proxy traits for WUE under LW conditions. Photoprotective and non-photochemical quenching traits (ΦNPQ, NPQt, Fv/Fm, ΦNO) had lower contributions (VIP< 1), suggesting a weaker direct link to δ¹³C-derived iWUE in this experimental context.

Importantly, both the effect-size analysis and the PLS-VIP results indicate that the imposed LW treatment did not induce severe physiological stress. Photoprotective traits such as NPQt, ΦNPQ, and ΦNO, typically upregulated under strong drought or photoinhibitory pressure, showed weak to moderate responsiveness, while Fv/Fm remained relatively stable. This is consistent with the experimental design, where water was restricted during the vegetative stage but not to the extent of inducing severe drought. Instead, the strongest responses occurred in stomatal and leaf-surface traits, suggesting that plants primarily adjusted water-use regulation and leaf surface properties rather than activating high-level photoprotective mechanisms.

### 3.12 Composite multi-trait index

To integrate multiple physiological and anatomical traits contributing to stomatal regulation and WUE, a Composite Multi-Trait Index (CMTI) was developed for each genotype. Traits were selected based on both Cohen’s *d* effect size analysis and PLS-VIP scores, ensuring inclusion of variables that were most responsive to water limitation and most predictive of δ¹³C variation. These selected traits (ΦPSII, Fv/Fm, g_s_, SD, ΦNPQ, ΦNPQt, and CEW were first normalized to a 0–1 scale and directionally adjusted according to their expected response under PW or LW conditions. The composite index for genotype *i* was then computed as:


$$CMTI_{i}=\frac{1}{n}\sum_{j=1}^{n}Trait_{\left(i,j\right)}^{norm}$$

where $Trait_{i,j}^{norm}$ is the normalized value of trait *j* for genotype *i*, and *n* is the number of traits included. Conceptually, the CMTI follows the logic of multi-trait composite indices used in stress physiology, such as the Drought Response Index (DRI), which is based on grain yield adjusted for potential yield and flowering time (Pantuwan et al., 2002; Ouk et al., 2006). However, the CMTI is trait-based and was specifically developed in this study to integrate physiological and anatomical responses to water limitation rather than yield alone.

Importantly, we emphasize that the CMTI is used here as an integrative summary framework rather than as a definitive classifier of tolerance. Trait normalisation and weighting necessarily involve analytical decisions, and therefore CMTI values are interpreted alongside independent physiological evidence rather than in isolation. Higher CMTI values reflect stronger adaptive adjustment, whereas lower values indicate relatively stable performance with limited trait deviation.

**Figure 7:**
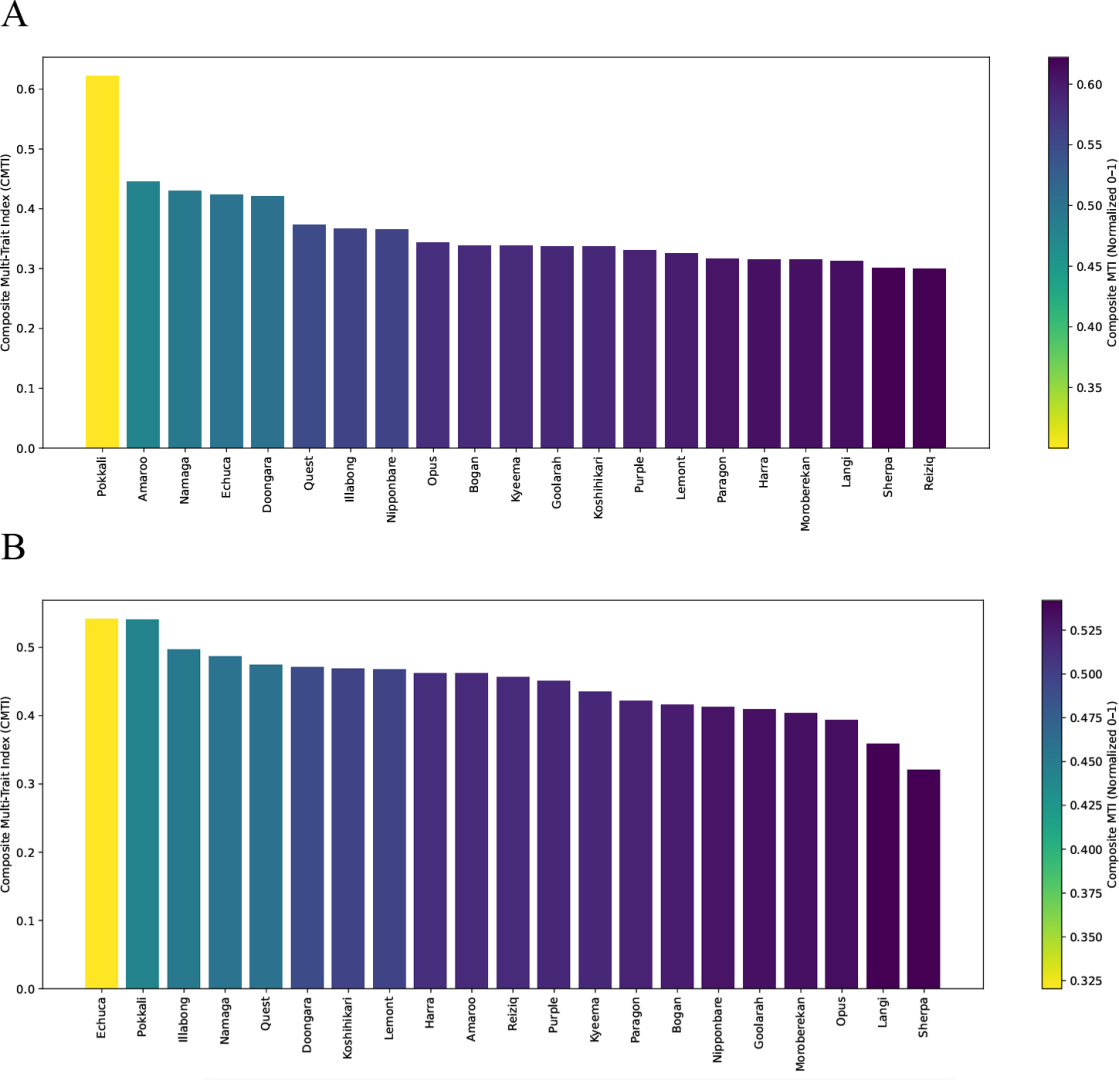
Composite MTI across rice genotypes evaluated under PW and LW based on **(A)** Cohen’s *d*–prioritised traits and **(B)** PLS-VIP–prioritised traits. Higher CMTI values (yellow and green) indicate stronger adaptive responses in key photosynthetic and anatomical traits under LW, while lower values (dark purple) represent stable performance and inherent tolerance.

When prioritising traits based on Cohen’s *d*, varieties such as Reiziq, Sherpa, Langi, Moroberekan, and Harra showed lower CMTI values, consistent with more stable physiological behaviour under both PW and LW conditions. In contrast, Pokkali, Amaroo, Namaga, Echuca, and Doongara exhibited higher CMTI values, indicative of stronger adaptive plasticity and pronounced trait adjustment in response to water limitation (**Figure 7A**).

When prioritising the traits identified by PLS-VIP, varieties such as Sherpa, Langi, Opus, and Moroberekan remained within the inherently stable group, while Echuca, Pokkali, Illabong, and Namaga clustered as varieties with strong adaptive plasticity and marked trait adjustment. The broad agreement between both prioritisation approaches strengthens confidence that these response patterns are biologically meaningful rather than artefacts of a single analytical method (**Figure 7B**).

Together, these contrasting patterns suggest that genotypes fall along a continuum from constitutive physiological stability to highly plastic stress adjustment. While we acknowledge that full validation will require independent assessment, including field-based testing and integration with additional agronomic traits, the CMTI provides a transparent and biologically grounded tool to summarise complex trait responses and support genotype prioritisation for future breeding which we hope to validate by population and genetics approaches in subsequent studies.

### 3.13 Mixed-effects model analysis

A linear mixed-effects model analysis was used to assess treatment, trial, and their interaction effects on physiological and surface traits (**Supplementary Table 2**). Water treatment significantly affected most traits, with NPQt, ΦNPQ, LT, and leaf CEW higher under LW, and ΦPSII, Fv/Fm, RCh, gs, and SD higher under PW. Trial effects, such as the slightly hotter conditions in T2, had minor influences, but the overall patterns of water treatment responses remained consistent. Significant treatment × trial interactions were observed for NPQt, LT, and SD, indicating slight variation in response magnitude between trials without altering the overall PW versus LW differences. **Supplementary Table 2** summarizes these effects, highlighting the traits most responsive to water treatment and their biological relevance.

### 3.14 Carbon isotope composition (δ¹³C) and leaf carbon–nitrogen traits

**Figure 8:**
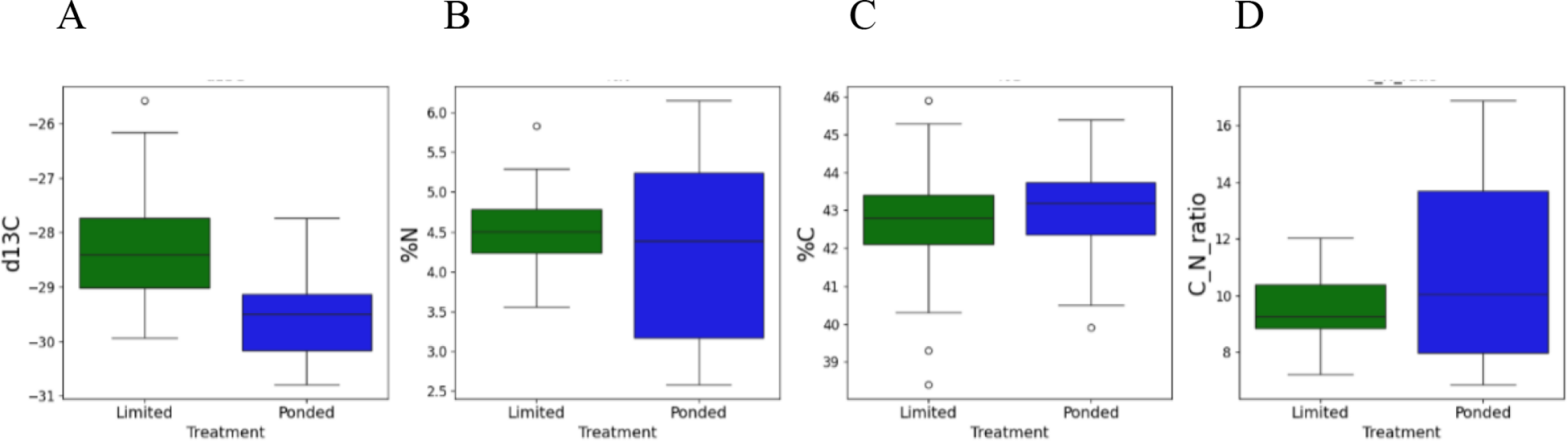
Leaf **(A)** δ¹³C, **(B)** %N, **(C)** %C, and **(D)** C:N ratio in limited water (LW, green) and ponded water (PW, blue) conditions.

As shown in **Figure 8**, leaf δ¹³C values were significantly lower (more negative) under PW conditions (-29.51 ± 0.85‰) compared to LW (-28.29 ± 1.03‰; t = 4.68, df = 52.66, p< 0.001), indicating that plants under LW exhibited higher intrinsic WUE (iWUE), this result is taken as an integrative indicator of gas-exchange balance, reflecting coordinated adjustments in stomatal behaviour and carbon assimilation under LW conditions. Leaf nitrogen concentration (%N) was slightly higher under LW (4.52 ± 0.53%) than under PW conditions (4.28 ± 0.73%), although the difference was not statistically significant (t = 1.00, p = 0.321). Carbon concentration (%C) remained similar between treatments (42.9 ± 1.5% under LW and 42.9 ± 1.7% under PW; t = -0.70, p = 0.485), indicating stable carbon accumulation regardless of water availability. The C:N ratio was lower under LW (9.55 ± 1.18) compared to PW conditions (10.88 ± 3.31; t = -2.02, p = 0.048), consistent with increased nitrogen investment in photosynthetic proteins and metabolic processes. Overall, these results suggest that water limitation strongly influences δ¹³C, reflecting intrinsic WUE adjustments, while leaf nitrogen and carbon contents are comparatively less responsive. The slightly lower C:N ratio under LW further supports enhanced metabolic allocation to photosynthetic machinery. **Table 2** highlights the top-performing rice varieties for each trait under LW conditions, providing a clear view of genotype-specific physiological and nutritional responses.

**Table 2:** Top six rice varieties with the largest responses in δ¹³C, %N, %C, and C:N ratio under limited (LW) vs ponded water (PW) conditions.

Parameter	Trend	Interpretation	Top varieties
δ¹³C	Less negative under LW	Higher intrinsic WUE of LW plants	Moroberekan, Harra, Sherpa, Purple, Nipponbare, Koshihikari, Langi
%N	Higher under LW	Increased leaf nitrogen allocation	Amaroo, Doongara, Goolarah, Namaga Sherpa, Moroberekan,
%C	Stable across treatments	Leaf carbon content largely unaffected	Doongara, Harra, Paragon, Sherpa, Moroberekan, Nipponbare,
C:N ratio	Lower under LW	Enhanced nitrogen allocation relative to carbon	Amaroo, Doongara, Namaga, Moroberekan, Sherpa, Goolarah

### 3.15 Whole-plant water use under limited-water conditions

Whole-plant irrigation demand under LW revealed clear and biologically meaningful differences among genotypes (**Supplementary Figure 12**). *Indica* varieties required the greatest total water input, consistent with their higher panicle numbers and greater tillering capacity, while among *japonica*, Kyeema exhibited the highest water requirement, aligning with its previously observed high cuticular water loss. Sherpa required comparatively less water under LW, which exhibited high epicuticular wax deposition and reduced cuticular water loss, indicating coordinated structural and physiological water-saving strategies. Importantly, several of these same varieties (e.g., Sherpa, Moroberekan, Harra, Nipponbare, Koshihikari and Langi) also showed less negative δ¹³C values under LW, supporting an interpretation of improved iWUE rather than simply reduced growth or transpiration. Together, the congruence between whole-plant water use, δ¹³C signatures, and leaf surface traits indicates that the observed variation reflects intrinsic physiological strategies under controlled moderate stress rather than unequal access to water.

### 3.16 Yield and reproductive performance measurements

**Figure 9:**
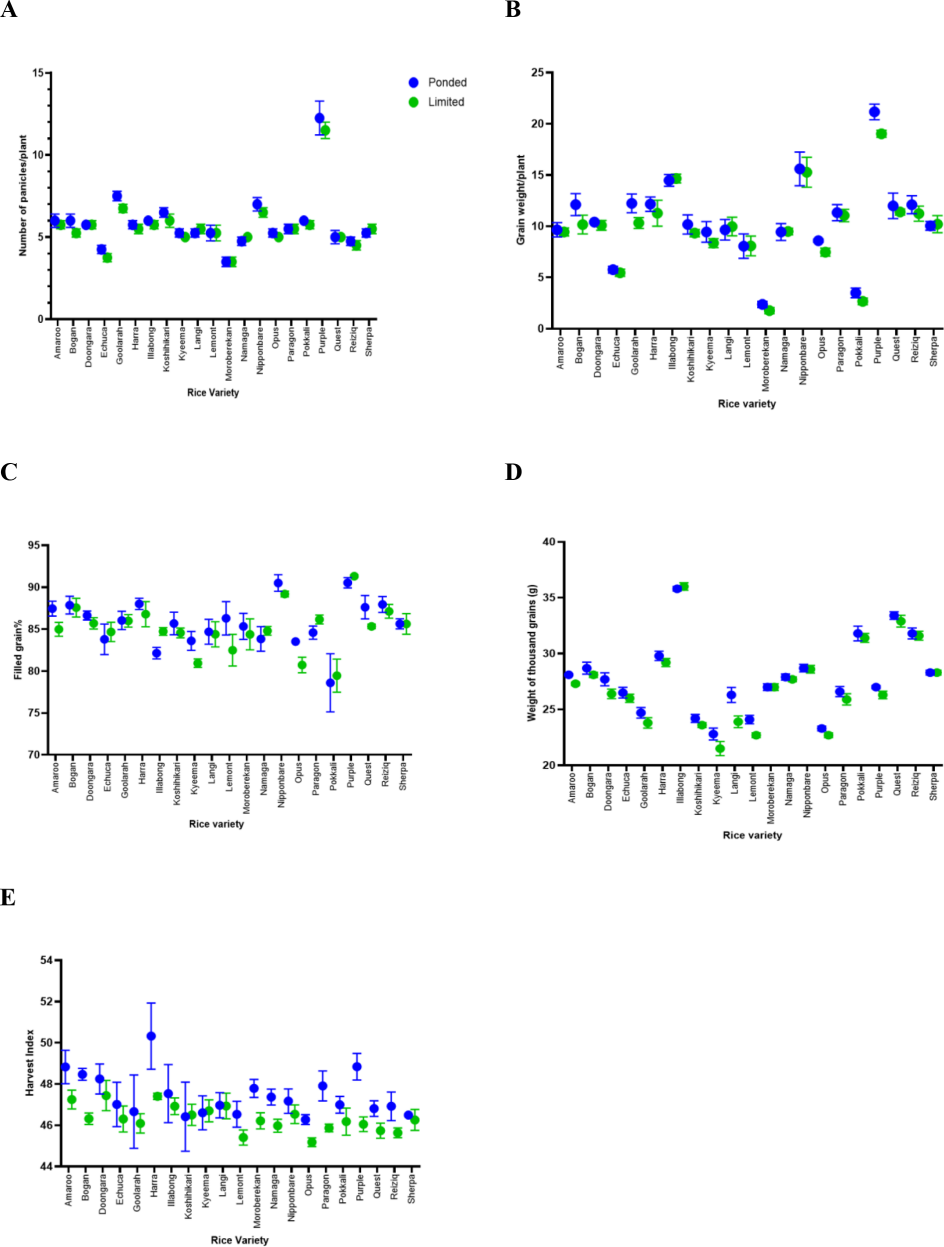
Effects of moderate limited-water treatment on reproductive and yield-related traits in rice. **(A)** Panicle number per plant, **(B)** total grain mass per plant, **(C)** filled grain percentage, **(D)** thousand-grain weight (TGW), and **(E)** harvest index (HI) in ponded water (PW) and limited water (LW) conditions. LW did not significantly affect panicle number (p = 0.700), grain mass per plant (p = 0.712), filled grain percentage (p = 0.771), or TGW (p = 0.877). HI showed a modest but significant reduction under LW (p = 0.043). Subspecies differences were detected only for panicle number (p< 0.001), with indica lines producing more panicles, while no treatment × subspecies interactions were detected for any trait, indicating comparable responses across genetic background. Data represent mean ± SEM, n = 21 varieties with 8 biological replicates per variety per treatment.

Moderate water limitation did not impose a reproductive penalty on the evaluated rice genotypes (**Figure 9**). LW supply did not significantly influence panicle number (p = 0.700) (**Figure 9**). However, subspecies differed significantly (p< 0.001), with indica lines (Purple produced the highest number of panicles) generally producing a greater number of panicles than japonica, consistent with known subspecies architectural distinctions. No treatment × subspecies interaction was detected (p = 0.824), indicating similar plasticity across backgrounds. Total grain mass per plant (**Figure 9**) was also unaffected by limited water (p = 0.712) and did not differ significantly between japonica and indica genotypes (p = 0.497), with no treatment × subspecies interaction (p = 0.827). Thus, total grain production per plant was preserved under moderate water limitation. Filled grain percentage remained stable under limited water (**Figure 9**), with no significant effect of treatment (p = 0.771), subspecies (p = 0.539), or their interaction (p = 0.586). These results indicate that spikelet fertility and successful grain filling were maintained despite reduced soil moisture availability. TGW remained unchanged under limited water (p = 0.877) (**Figure 9**) and did not differ between subspecies (p = 0.629), with no interaction effect (p = 0.947), demonstrating that individual grain size was stable across treatments. Harvest index exhibited a modest but statistically significant treatment effect (p = 0.043), with plants under limited water (**Figure 9**) showing a small reduction in HI relative to ponded controls. This indicates a slight shift in biomass partitioning toward vegetative tissues under LW, although subspecies differences (p = 0.412) and the treatment × subspecies interaction (p = 0.400) were not significant, demonstrating a consistent response across genetic backgrounds.

Collectively, these results demonstrate that maintaining soil moisture at 60-65% FC induced meaningful physiological responses without compromising reproductive success. Grain filling, grain mass per plant, grain size, and panicle production were preserved, and only a small shift in harvest index suggested modest adjustment in biomass allocation rather than yield loss. These findings confirm that the imposed stress level represents moderate, agronomically relevant water limitation, supporting the interpretation of leaf-level physiological and anatomical responses in an agronomically meaningful context.

### 3.17 Grain milling quality

**Figure 10:**
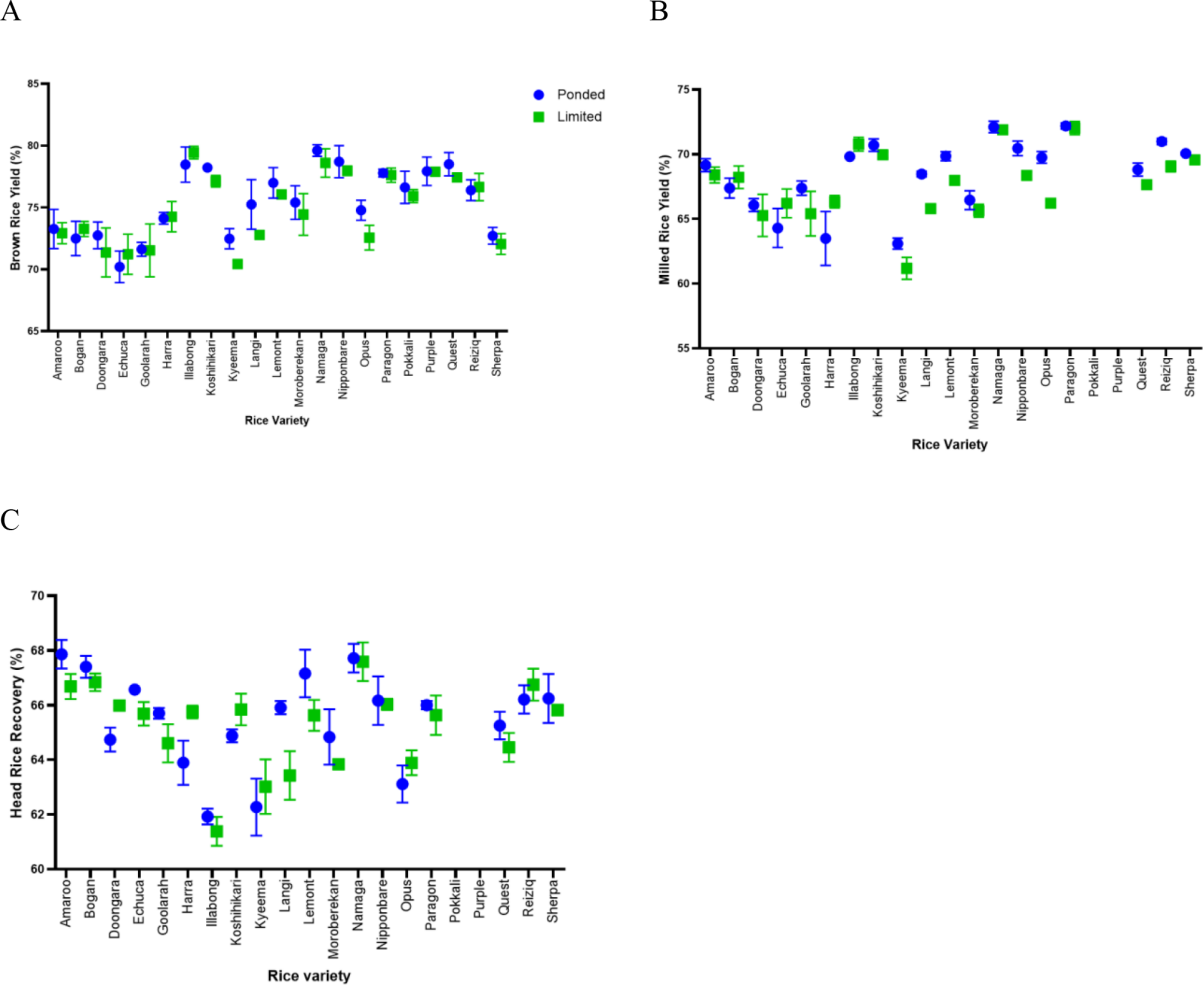
Grain milling quality of rice varieties grown under ponded (PW) and limited-water (LW) conditions. **(A)** Brown rice yield (BRY) was not significantly affected by water treatment (two-way ANOVA, Water p = 0.131; Subsp p = 0.303; Water × Subsp p = 0.699). **(B)** Milled rice yield (MRY) was slightly but significantly reduced under LW (Water p = 0.005). Only japonica genotypes were included in the MRY analysis because coloured indica lines were not polished. **(C)** Head rice recovery (HRR) did not differ significantly between PW and LW (Water p = 0.195). Data represent mean ± SEM, n = 21 varieties with 8 biological replicates. n = 21 genotypes for BRY; n = 19 japonica genotypes for MRY and HRR).

Water limitation during the vegetative stage did not significantly affect brown rice yield (BRY) (Water, p = 0.131) (**Figure 10**). Subspecies differences were also non-significant (p = 0.303), and no Water × Subspecies interaction was detected (p = 0.699), indicating that both japonica and indica genotypes maintained comparable dehusking efficiency under LW and PW conditions. BRY remained high across all genotypes, with mean values of 74.6–77.3% across treatments.

In contrast, milled rice yield (MRY) was slightly but significantly reduced under LW (Water, p = 0.005), with LW plants showing on average a 0.8–1.0% decrease compared with ponded controls (**Figure 10**). This effect was consistent across japonica genotypes included in the milling assessment, with no evidence of differential subspecies response because indica lines were not included due to coloured pericarp and non-polishable grain.

Head rice recovery (HRR) was not significantly affected by water treatment (Water, p = 0.195), with mean HRR values remaining stable between 65.2% and 65.5% under LW and PW (**Figure 10**). This indicates that grain structural integrity and resistance to breakage during milling were maintained despite moderate water limitation during vegetative growth.

Overall, these results demonstrate that maintaining soil moisture at 60-65% FC during vegetative growth did not compromise key milling quality traits, with BRY and HRR remaining unaffected and only a small decrease observed in MRY. These findings support the conclusion that the imposed LW regime represents a moderate and agronomically realistic level of stress that does not adversely impact postharvest rice processing performance.

## 4 Discussion

Rice adapts to water scarcity through a combination of morphological, physiological, genetic, and molecular mechanisms. Stress-resilient plants may avoid drought through architectural modification or tolerate it via physiological and biochemical adjustment, depending on the developmental stage and the severity and duration of water limitation (Fernando et al., 2025; Hussain et al., 2022, Hussain et al., 2020; Islam et al., 2009). Previous studies have shown that imposing drought at different stages of the rice life cycle, particularly when prolonged, can negatively impact both yield and grain quality (Baisakh et al., 2020; Kamoshita et al., 2015; Lafitte et al., 2007). Breeding for improved WUE under LW conditions is therefore essential to maintain productivity and grain quality. In this study, the LW treatment (60-65% of the original field capacity) closely aligns with Reddy Hussain et al (2020), although their treatment was applied only from 45 to 80 days after sowing rather than throughout the vegetative stage. By contrast, most previous studies imposed substantially more severe stress (<50% FC), often resulting in stronger adverse effects (Lafitte et al., 2007; Pitaloka et al., 2021; Praba et al., 2009; Siopongco et al., 2006).

Although the experiments were conducted in a glasshouse, meshed pot bases allowing root extension beyond containers, wide pot spacing, and continuous soil-moisture control helped minimise typical pot-related artefacts in rooting volume and water availability. However, we recognise that field hydraulic environments cannot be fully replicated under controlled conditions; therefore, these findings should be interpreted as mechanistic evidence that now warrants validation under multi-environment field trials. Future studies would benefit from direct measurement of leaf water potential to complement soil-based moisture monitoring as well as using a diversity panel for population level validation.

Previous studies have reported that g_s_ in rice under drought stress can vary between 30 and 150 mmol m^-^² s^-^¹, whereas well-watered plants typically exhibit g_s_ between 280 and 500 mmol m^-^² s^-^¹ (Farooq et al., 2010; Ouyang et al., 2017; Dien et al., 2017). In the present study, LW-treated plants maintained gs above 200 mmol m^-^² s^-^¹, indicating a mild stress condition that still allowed efficient gas exchange for sufficient carbon assimilation while minimising excessive water loss, which closely mimics optimal Australian rice growing conditions. PW plants, in contrast, represent well-watered conditions. The variety “Moroberekan”, previously reported as drought-tolerant (Grondin et al., 2018), was included here as a reference for WUE.

Reduced number of stomata conserves water but also limits carbon assimilation, requiring careful management (Caine et al., 2019). Moderate drought conditions contribute to a gradual decrease in stomata numbers, while severe drought leads to a more significant reduction (Ilyas et al., 2021). In the current study, LW plants had stomatal densities of more than 300/mm², higher than those typically seen in drought conditions, which is around 250/mm² (Freeg et al., 2022), suggesting improved yields. Caine et al. (2019) found that increased CO_2_ and higher temperatures could enable lower stomatal density varieties to maintain sufficient gas exchange and achieve equivalent or improved yields, even with reduced photosynthesis. Thus, slightly reducing stomatal density may potentially not negatively impact rice yields in the future with rising CO_2_ and temperatures.

Literature on rice leaf papillae is very limited, and to our knowledge (Pitaloka et al., 2021; Siregar et al., 2024), the number of papillae has not previously been quantified in rice leaves. In the present study, LW treated leaves developed larger papillae positioned over stomatal complexes. Although direct measurements of stomatal aperture dynamics were not undertaken, the close spatial association between enlarged papillae and stomata, together with reduced leaf water loss, is consistent with a potential moderating role in evaporative flux. In practical terms, LW plants showed reduced cuticular water loss and greater surface barrier reinforcement compared with PW leaves, indicating that papilla enlargement contributes meaningfully to water-saving function rather than representing a purely anatomical change. Thus, papillae enlargement is best interpreted as a plausible structural component of rice water-conservation strategies under moderate water limitation, warranting further functional validation.

Previous studies have shown that rice leaves produce more CEW under water stress (Haque et al., 1992; Srinivasan et al., 2008), but little is known about the role of wax papillae in managing stresses (Pitaloka et al., 2021). The larger papillae that cover the stomata, observed on the LW plant leaves in this study, might be a key adaptation that helps rice leaves conserve water and protect against various abiotic and maybe biotic stresses. The enlargement of stomatal papillae observed under LW conditions likely represents a critical compensatory adaptation for maintaining WUE, particularly within the context of modern breeding preferences. Most Australian commercial varieties carry the recessive allele for glabrousness (smooth leaves), a trait selected to eliminate silica-rich trichomes (leaf hairs) that cause mechanical injury and ‘rice itch’ during harvest. This smooth-leaf phenotype likely entered Australian germplasm via introgression from the United States, specifically tracing back to Arkansas and Southern USA breeding lines, where the glabrous trait is predominant. In the absence of pubescence (hairs) to physically trap a humid boundary layer, these glabrous varieties must rely on alternative mechanisms, such as enhanced glaucousness (epicuticular wax) and the micro-turbulence generated by larger papillae, to reduce evapotranspiration and protect against abiotic stress. On the other hand, flavonoids help plants cope with water stress by acting as antioxidants, scavenging reactive oxygen species (ROS), and protecting against UV damage. They also regulate stress-related hormones, maintain osmotic balance, and enhance root development, improving the overall drought tolerance of the plant (Laoué et al., 2022; Shomali et al., 2022). The results of the current study show that flavonols play a promising role in stress tolerance in rice leaves exposed to LW regimes. Moreover, the results of this study indicate that as LW leaves reach the optimal wax level at an increasing rate, they produce more flavonols than PW leaves.

The reduction in ΦPSII and the corresponding increase in ΦNPQ observed in LW plants were relatively modest compared with previous severe drought studies (Caine et al., 2019; Pitaloka et al., 2022). This pattern indicates that photochemical efficiency was largely maintained and that photoprotective energy dissipation was activated without imposing substantial photoinhibition, consistent with expectations for mild water limitation. These results align with Vijayaraghavareddy et al. (2022), who reported similar shifts in ΦPSII and ΦNPQ under LW conditions at approximately 60% FC. Collectively, this suggests that the stress imposed in the present study is within a physiological range that allows rice plants to preserve effective photosynthetic electron transport while enhancing regulated energy dissipation, an adaptive balance likely contributing to stable carbon assimilation and potential yield maintenance under LW.

Measuring the contact angle of water on rice leaves under contrasting water regimes provides important insights into surface hydrophobicity, epicuticular wax expression, and their roles in water conservation. In many species, water limitation leads to increased deposition or restructuring of epicuticular waxes, resulting in higher contact angles and reduced cuticular transpiration (Papierowska et al., 2018). In the present study, however, genotype-specific responses were evident, while some varieties (e.g., Amaroo, Bogan etc) showed little or no change in contact angle under LW, they exhibited strong physiological adjustments such as reduced g_s_, suggesting alternative drought-response strategies driven by stomatal traits rather than surface hydrophobicity. In contrast, other genotypes increased their contact angle under LW, consistent with enhanced CEW deposition as a protective mechanism. These contrasting patterns highlight that rice genotypes employ different combinations of surface and stomatal adaptations to regulate water loss. It is also important to note the practical limitations associated with contact-angle measurements in rice. Many leaves displayed curvature, undulating surfaces, or surface trichomes, making it challenging to identify sufficiently flat regions for accurate droplet placement. These structural constraints likely restricted the range of measurable differences among varieties. Nevertheless, the results demonstrate that contact angle remains a useful, though genotype-sensitive, indicator of surface hydrophobicity and can complement anatomical and physiological traits when evaluating WUE-related adaptations. Previous studies highlight the importance of δ¹³C as an integrative indicator of intrinsic water-use efficiency in C_3_ plants, reflecting coordinated adjustments in stomatal conductance and carbon assimilation (Wang et al., 2022; Jiang et al., 2024). Consistent with this framework, δ¹³C patterns in the present study aligned closely with trait-based evidence from stomatal regulation, wax deposition, cuticular water-loss behaviour, papillae enlargement, and whole-plant water use, together supporting the interpretation that the observed responses represent coordinated WUE strategies rather than artefacts of differential water access.

While subspecies contrasts were explicitly tested for stomatal traits, δ¹³C, and yield components, they were not statistically evaluated for PhotosynQ fluorescence traits, leaf surface hydrophobicity, or whole-plant water use. Therefore, differences described for these latter traits are interpreted at the genotype level rather than as confirmed japonica–indica contrasts. These patterns are biologically consistent with known subspecies physiology, but we caution against over-generalisation without formal subspecies testing and multi-environment or multi-season validation. We hope to address this limitation in subsequent studies.

Moderate LW did not negatively affect key grain quality traits. Brown rice yield and head rice recovery were maintained, and only a small reduction in MRY was detected, without compromising marketable grain quality. These results support LW as a moderate, agronomically relevant stress level with minimal milling quality penalty. Importantly, unlike many severe drought studies that report strong reproductive penalties, the carefully controlled LW regime applied here elicited adaptive responses without substantial loss of reproductive performance, a scenario highly relevant to irrigated Australian production systems where the goal is increasingly to optimise rather than maximise water inputs. Taken together, these findings indicate that targeted combinations of physiological and structural traits can support improved WUE under moderate water constraints, while acknowledging that multi-environment and multi-season field validation remains essential before translation into breeding practice.

## 5 Conclusion

This study reveals that rice genotypes achieve water use efficiency through two distinct mechanisms under moderate vegetative-stage water limitations. By maintaining field capacity at 60–65% rather than imposing severe drought, we identified adaptive responses that conserve water without severely compromising photosynthetic performance. Genotypes segregated into inherently tolerant types (Moroberekan, Sherpa, Harra, Reiziq, Langi, Paragon) with stable physiological performance, and adaptive types (Pokkali, Doongara, Namaga, Amaroo, Echuca) exhibiting pronounced phenotypic plasticity. The most significant structural finding was substantial papillae enlargement on leaf surfaces under water limitation, with apex area increasing approximately 20% and physically occluding stomatal pores. This adaptation, coupled with enhanced cuticular wax deposition and increased surface hydrophobicity, provides a critical water conservation mechanism for smooth-leaf Australian varieties bred without protective trichomes. Carbon isotope discrimination validated superior intrinsic water use efficiency in limited water-treated plants, confirming the physiological and structural trait responses. These complementary strategies provide multiple breeding pathways for improving water use efficiency without a yield penalty. The identified physiological markers, particularly papillae morphology, stomatal density, and wax deposition, offer practical selection criteria for developing water-efficient temperate rice varieties adapted to Australian production systems under increasingly variable water availability.

## Data Availability

The original contributions presented in the study are included in the article/**Supplementary Material**. Further inquiries can be directed to the corresponding author.

## Supplementary material

Supplementary Figure 1 Soil dry-down curve for the potting mix used in the experiment. Plants showed drought resistance response (leaf drooping) on day 7, indicating the wilting point. The moisture content on day 4 (25-28%) was selected for maintaining limited water conditions, corresponding to 60-65% of field capacity.

Supplementary Figure 2 Stomatal conductance (g_s_) of rice varieties under ponded (PW, blue) and limited water (LW, green) conditions. All varieties showed reduced g_s_ under LW. *Indica* had higher g_s_ than *japonica* across treatments (LW *indica* 355 ± 50 mmol m^-^² s^-^¹> *japonica* 312 ± 53 mmol m^-^² s^-^¹ > PW *indica* 442 ± 59 mmol m^-^² s^-^¹ > *japonica* 422 ± 86 mmol m^-^² s^-^¹), with no significant treatment × subspecies interaction. Variety-specific differences highlight genotypic variation in stomatal response to water limitation.

Supplementary Figure 3 Abaxial stomatal density (SD) of rice varieties under ponded (PW, blue) and limited water (LW, green) conditions. All varieties showed reduced SD under LW. *Indica* had higher SD than *japonica* across treatments (LW *indica* 427 ± 7 mm^-^²< *japonica* 377 ± 32 mm^-^²< PW *indica* 510 ± 12 mm^-^²< *japonica* 439 ± 29 mm^-^²), with no significant treatment × subspecies interaction. Variety-specific differences reflect genotypic variation in potential gas exchange and water-use strategies.

Supplementary Figure 4 ATR-FTIR spectra of fresh rice leaves from ponded water (PW, blue) and limited water (LW, green) plants. Key peaks identified include those for cuticular and epicuticular waxes (2800–3000 cm^-^¹) and flavonols (1125–1140 cm^-^¹, 1205–1225 cm^-^¹, 1270–1310 cm^-^¹, 1435–1475 cm^-^¹, and 1605–1620 cm^-^¹). Representative spectra shown are from Sherpa variety with comparable spectra for other varieties tested.

Supplementary Figure 5 Papillae density on leaf surfaces of 21 rice varieties grown under ponded water (PW, blue) and limited water (LW, green) conditions. Papillae counts were obtained from 12 SEM images per variety, derived from four leaves per genotype. Papillae number varied significantly among varieties (two-way ANOVA, p< 0.001), with a marginal effect of water treatment (p = 0.064) and no significant variety × treatment interaction (p = 0.999), indicating comparable responses under PW and LW. Indica varieties exhibited higher papillae density than Japonica varieties (p< 0.001), irrespective of water regime.

Supplementary Figure 6 Papilla apex area (µm²) on the adaxial surface of rice leaves under ponded (PW, blue) and limited water (LW, green) conditions. Measurements were derived from SEM images using ImageJ (n = 21 varieties; 12 replicate images from 4 leaves per variety). Papilla apex area differed significantly among varieties (two-way ANOVA, p< 0.001), with strong effects of water treatment (p< 0.001) and a significant variety × treatment interaction (p< 0.001). Moroberekan exhibited the largest papilla apex area under PW, and under LW most varieties showed increased papillae area, with Moroberekan and Sherpa showing the largest values. No significant effect of subspecies or treatment × subspecies interaction was observed.

Supplementary Figure 7 Leaf water loss (%) under ponded (PW, blue) and limited water (LW, green) conditions. LW leaves showed lower water loss than PW leaves. Moroberekan and Sherpa exhibited the lowest water loss, while Kyeema showed the highest. Reduced water loss corresponds with higher epicuticular wax on Moroberekan and Sherpa leaf surfaces.

Supplementary Figure 8 The contact angle of ponded water (PW) and limited water (LW) plant leaves at Wk10. Data represent the mean ± SE of 6 measurements per variety, obtained from three plants per variety, with two measurements taken from each plant. p-value (t-test)<0.001

Supplementary Figure 9 Triangular correlation matrices of rice physiological and structural traits under P **(A)** and L **(B)** water treatments (green increasing positive correlation; purple increasing negative correlation).

Supplementary Figure 10 Cohen’s d effect sizes comparing ponded (PW) and limited water (LW) treatments for measured physiological and structural traits.

Supplementary Figure 11 Variable Importance in Projection (VIP) scores from Partial Least Squares (PLS) regression using δ¹³C as the response variable. Traits with VIP ≥ 1 are identified as strong contributors explaining variation in δ¹³C, while traits with VIP< 1 are considered weak predictors.

Supplementary Figure 12 Whole-plant water use under limited-water (LW) conditions. Genotypes differed significantly in cumulative irrigation requirement, reflecting biological variation in canopy water use and physiological regulation under LW. Indica varieties generally required greater water inputs, consistent with higher tiller number, whereas Sherpa exhibited more conservative water use. These patterns align with leaf trait behaviour and δ¹³C responses, supporting genuine variation in intrinsic WUE. Data represent mean ± SEM, n = 21 varieties with 8 biological replicates.

Supplementary Table 1 Twenty-one (21) rice lines provided by the NSW Department of Primary Industries and Regional Development, New South Wales, Australia. These included eighteen Australian temperate *japonica* commercial rice lines, two *indica* rice varieties and Moroberekan (a hybrid variety of *japonica* and *indica*) as the positive control.

Supplementary Table 2 Summary of mixed-effects model results for physiological traits across two trials and treatments: ponded (P) and limited (L). Significance codes: ns = not significant, *p< 0.05, **p< 0.01, ***p< 0.001
