# Supplementary material for: Divergent physiological strategies distinguish tolerant and plastic genotypes in elite Australian rice lines under limited irrigation
Source: Front Plant Sci. 2026 Feb 13;17:1760397. doi: 10.3389/fpls.2026.1760397 (PMC12947703; doi:10.3389/fpls.2026.1760397)
Supplement: Supplementary Figure 1 — Soil dry-down curve for the potting mix used in the experiment. Plants showed drought resistance response (leaf drooping) on day 7, indicating the wilting point. The moisture content on day 4 (25-28%) was selected for maintaining limited water conditions, corresponding to 60-65% of field capacity. [file DataSheet1.docx]

Supplementary Material

# Supplementary Data

# Supplementary Figures and Tables

## Supplementary Tables

**Supplementary Table 1**. Twenty-one (21) rice lines provided by the NSW Department of Primary Industries and Regional Development, New South Wales, Australia. These included eighteen Australian temperate *japonica* commercial rice lines, two *indica* rice varieties and Moroberekan (a hybrid variety of *japonica* and *indica*) as the positive control.

| **Rice variety** | **Subspecies** | **Grain classification** |
| --- | --- | --- |
| Amaroo | *Japonica* | Medium grain |
| Bogan | *Japonica* | Medium grain |
| Doongara | *Japonica* | Long grain |
| Echuca | *Japonica* | Medium grain |
| Goolarah | *Japonica* | Fragrant |
| Harra | *Japonica* | Arborio |
| Illabong | *Japonica* | Arborio |
| Koshihikari_(Y4) | *Japonica* | Short grain |
| Kyeema | *Japonica* | Fragrant |
| Langi_(Y1) | *Japonica* | Long grain |
| Lemont_(Y2) | *Japonica* | Long grain |
| Moroberekan_(Y2) | *Japonica* | Short grain |
| Namaga | *Japonica* | Medium grain |
| Nipponbare_(Y3) | *Japonica* | Medium grain |
| Opus | *Japonica* | Short grain |
| Paragon | *Japonica* | Medium grain |
| Pokkali | *Indica* | Medium grain |
| Purple | *Indica* | Coloured Long grain |
| Quest CT-18 | *Japonica* | Medium grain |
| Reiziq | *Japonica* | Medium grain |
| Sherpa | *Japonica* | Medium grain |

**Supplementary Table 2.** Summary of mixed-effects model results for physiological traits across two trials and treatments: ponded (P) and limited (L). Significance codes: ns = not significant, * p < 0.05, ** p < 0.01, *** p < 0.001

| **Trait** | **Treatment**  **(p-value)** | **Trial**  **(p-value)** | **Treatment × Trial**  **(p-value)** | **Key Observation / Biological Insight** |
| --- | --- | --- | --- | --- |
| NPQt | *** <0.001 | *** <0.001 | *  0.014 | Higher in L overall; slightly lower in T2 |
| Phi2 | *** <0.001 | *** <0.001 | ns  0.680 | Higher in P; T2 > T1 |
| PhiNPQ | *** <0.001 | *** <0.001 | ns  0.981 | Higher in L; T1 > T2 |
| RCh | *** <0.001 | *** <0.001 | *  0.029 | Higher in P; slightly stronger difference in T2 |
| Fv/Fm | *** <0.001 | *** <0.001 | ns  0.359 | Higher in P; T2 > T1 |
| LT | *** <0.001 | *** <0.001 | *** <0.001 | Higher in L; strong trial-dependent differences |
| Wax | *** <0.001 | *** <0.001 | ns  0.149 | Higher in L; slight T2 > T1 |
| SDL | *** <0.001 | *** <0.001 | **  0.017 | Higher in P; stronger P effect in T2 |
| SC | *** <0.001 | *** <0.001 | **  0.02 | Higher in P; stronger P effect in T2 |

## Supplementary Figures


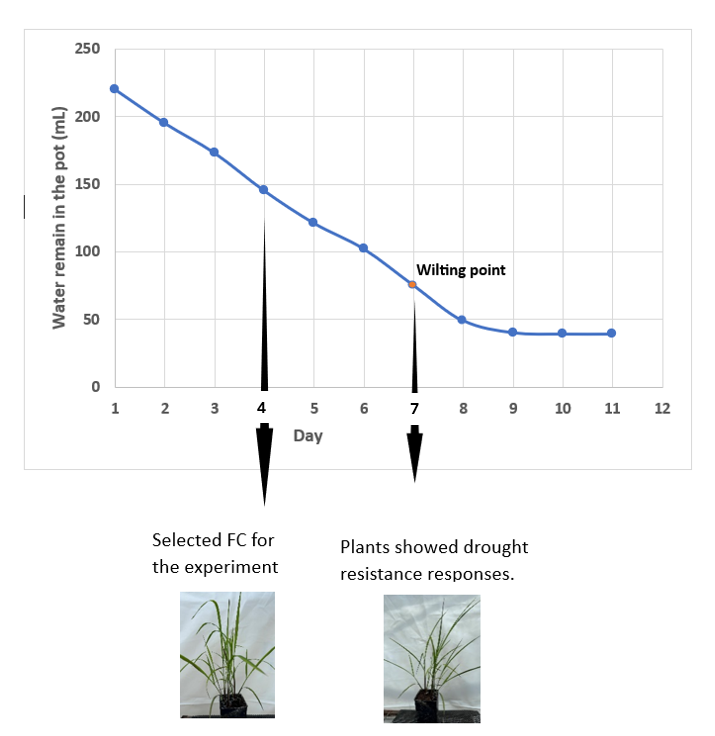


**Supplementary Figure 1.** Soil dry-down curve for the potting mix used in the experiment. Plants showed drought resistance response (leaf drooping) on day 7, indicating the wilting point. The moisture content on day 4 (25-28%) was selected for maintaining limited water conditions, corresponding to 60-65% of field capacity.


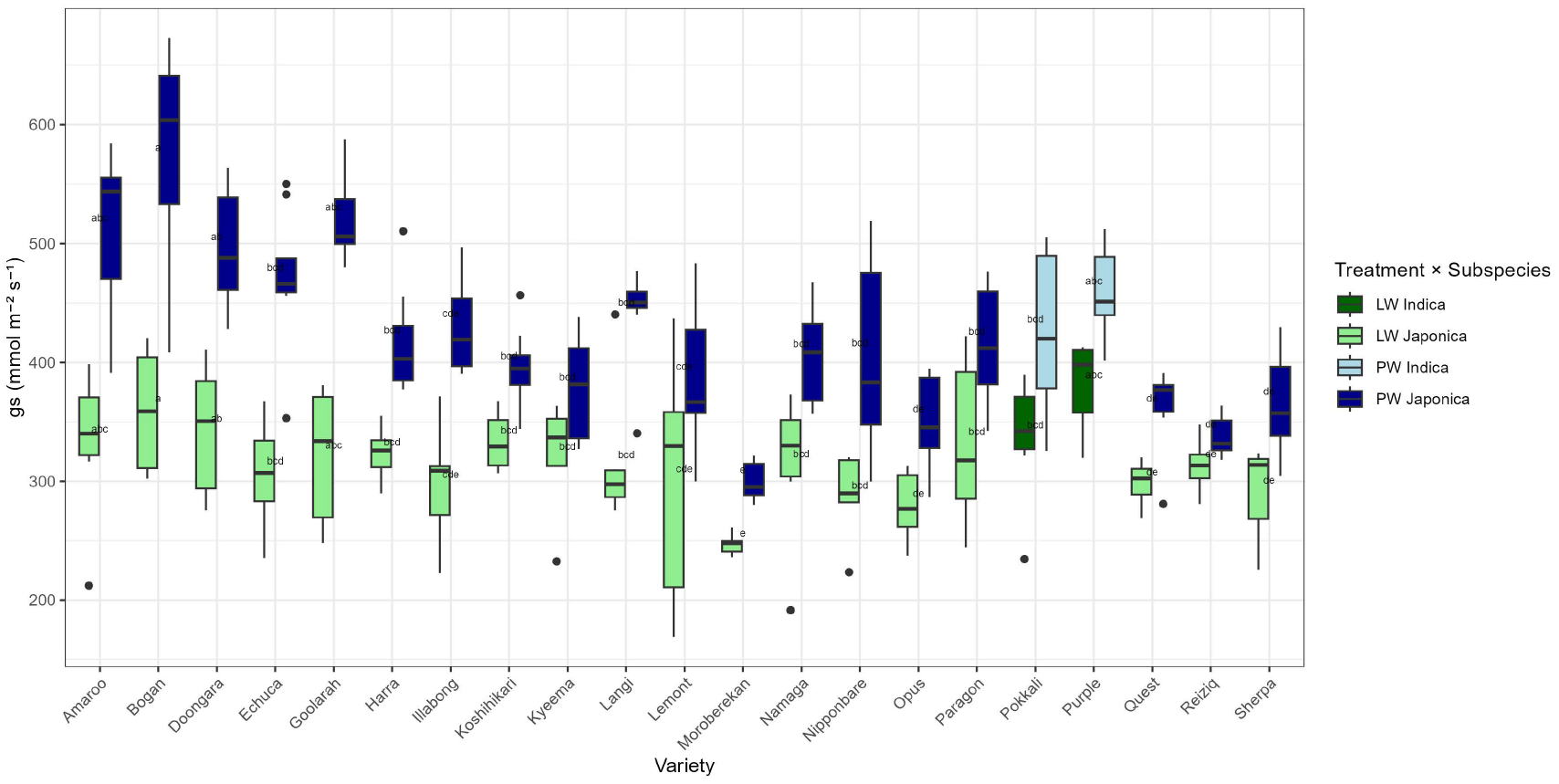


**Supplementary Figure 2**. Stomatal conductance (g_s_) of rice varieties under ponded (PW, blue) and limited water (LW, green) conditions. All varieties showed reduced g_s_ under LW. *Indica* had higher g_s_ than *japonica* across treatments (LW *indica* 355 ± 50 mmol m⁻² s⁻¹> *japonica* 312 ± 53 mmol m⁻² s⁻¹ > PW *indica* 442 ± 59 mmol m⁻² s⁻¹ > *japonica* 422 ± 86 mmol m⁻² s⁻¹), with no significant treatment × subspecies interaction. Variety-specific differences highlight genotypic variation in stomatal response to water limitation.


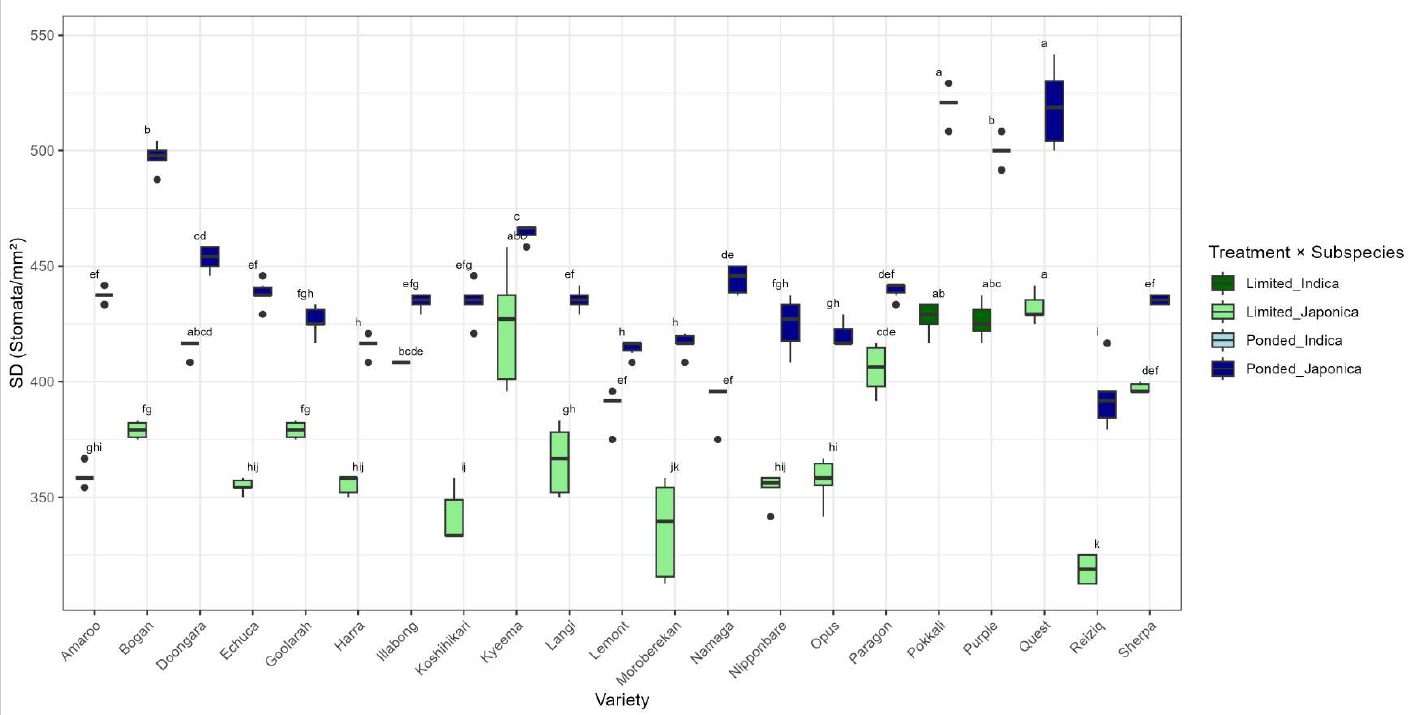


**Supplementary Figure 3.** Abaxial stomatal density (SD) of rice varieties under ponded (PW, blue) and limited water (LW, green) conditions. All varieties showed reduced SD under LW. *Indica* had higher SD than *japonica* across treatments (LW *indica* 427 ± 7 mm⁻² < *japonica* 377 ± 32 mm⁻² < PW *indica* 510 ± 12 mm⁻² < *japonica* 439 ± 29 mm⁻²), with no significant treatment × subspecies interaction. Variety-specific differences reflect genotypic variation in potential gas exchange and water-use strategies.


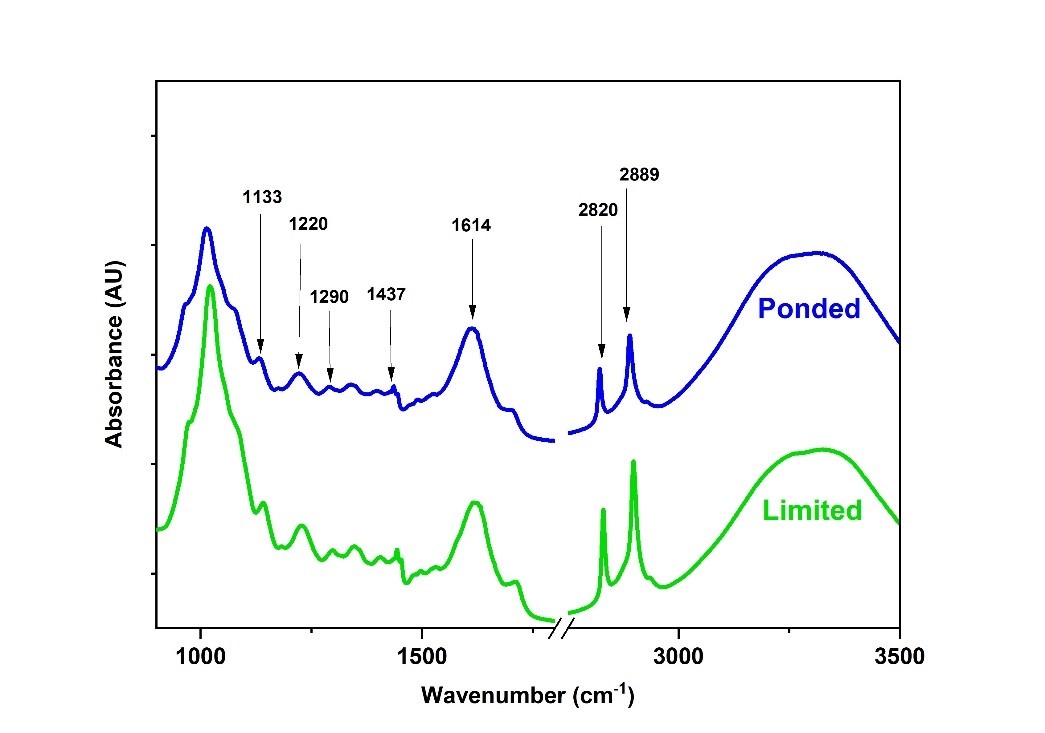


**Supplementary Figure 4.** ATR-FTIR spectra of fresh rice leaves from ponded water (PW, blue) and limited water (LW, green) plants. Key peaks identified include those for cuticular and epicuticular waxes (2800-3000 cm⁻¹) and flavonols (1125-1140 cm⁻¹, 1205-1225 cm⁻¹, 1270-1310 cm⁻¹, 1435-1475 cm⁻¹, and 1605-1620 cm⁻¹). Representative spectra shown are from Sherpa variety with comparable spectra for other varieties tested.


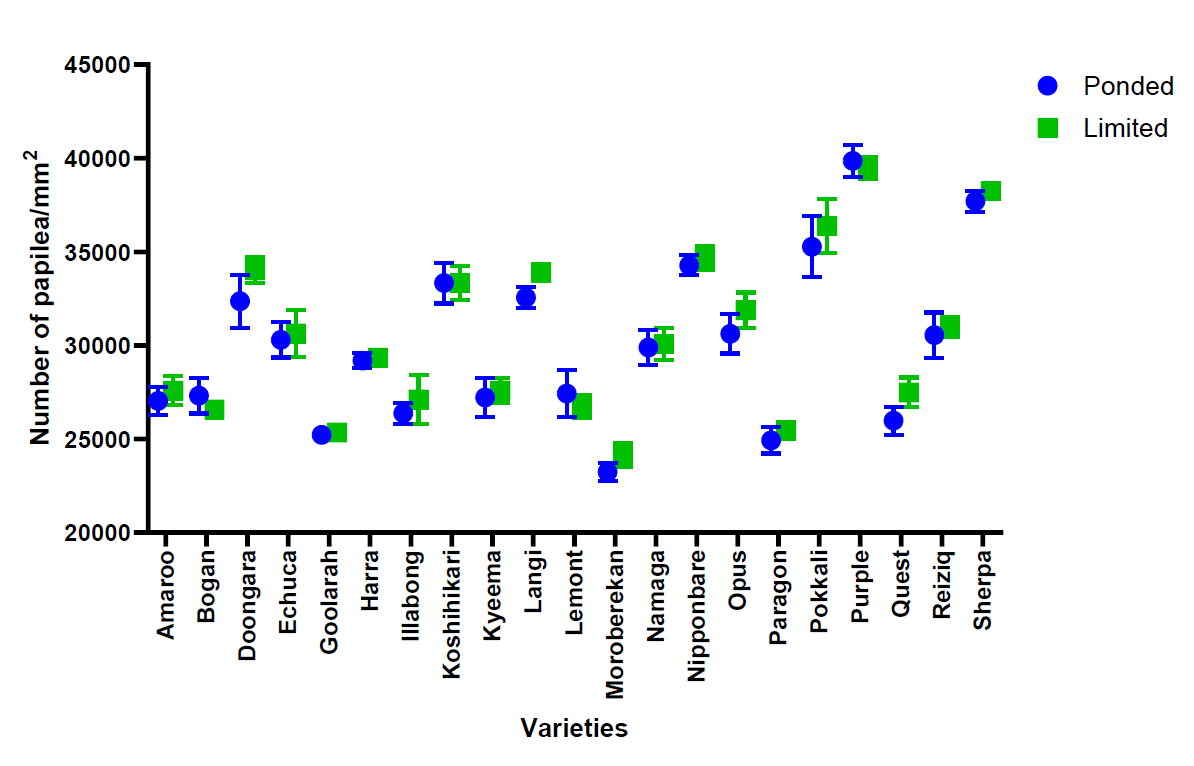


**Supplementary Figure 5.** Papillae density on leaf surfaces of 21 rice varieties grown under ponded water (PW, blue) and limited water (LW, green) conditions. Papillae counts were obtained from 12 SEM images per variety, derived from four leaves per genotype. Papillae number varied significantly among varieties (two-way ANOVA, p < 0.001), with a marginal effect of water treatment (p = 0.064) and no significant variety × treatment interaction (p = 0.999), indicating comparable responses under PW and LW. Indica varieties exhibited higher papillae density than Japonica varieties (p < 0.001), irrespective of water regime.


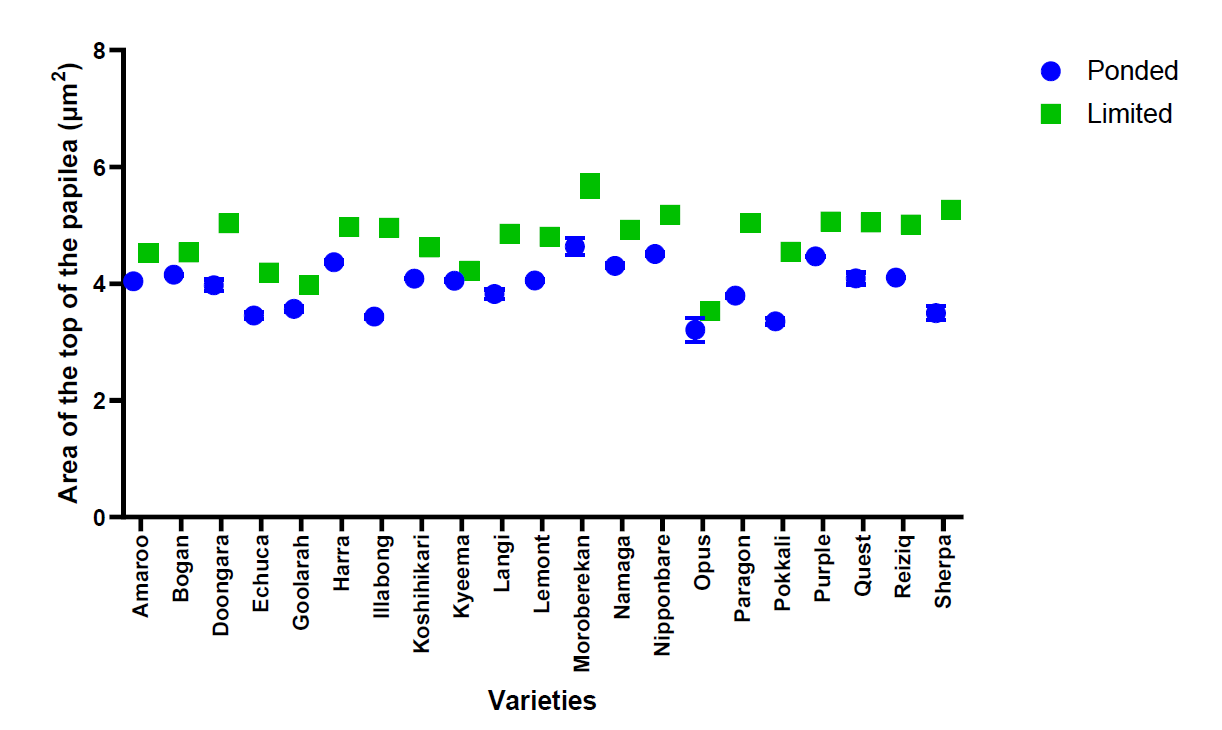


**Supplementary Figure 6.** Papilla apex area (µm²) on the adaxial surface of rice leaves under ponded (PW, blue) and limited water (LW, green) conditions. Measurements were derived from SEM images using ImageJ (n = 21 varieties; 12 replicate images from 4 leaves per variety). Papilla apex area differed significantly among varieties (two-way ANOVA, p < 0.001), with strong effects of water treatment (p < 0.001) and a significant variety × treatment interaction (p < 0.001). Moroberekan exhibited the largest papilla apex area under PW, and under LW most varieties showed increased papillae area, with Moroberekan and Sherpa showing the largest values. No significant effect of subspecies or treatment × subspecies interaction was observed.


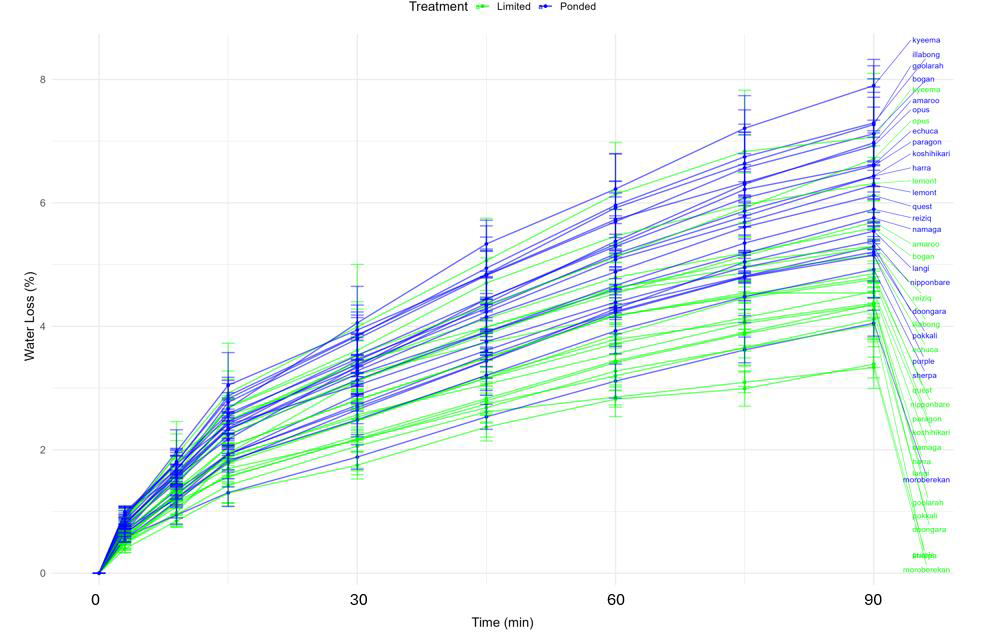


Supplementary Figure 7. Leaf water loss (%) under ponded (PW, blue) and limited water (LW, green) conditions. LW leaves showed lower water loss than PW leaves. Moroberekan and Sherpa exhibited the lowest water loss, while Kyeema showed the highest. Reduced water loss corresponds with higher epicuticular wax on Moroberekan and Sherpa leaf surfaces.


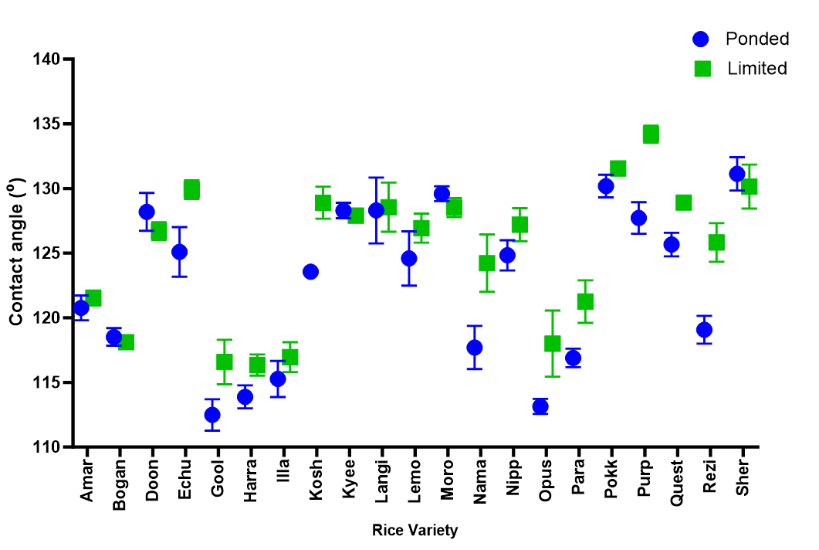
Supplementary Figure 8. The contact angle of ponded water (PW) and limited water (LW) plant leaves at Wk10. Data represent the mean ± SE of 6 measurements per variety, obtained from three plants per variety, with two measurements taken from each plant. p-value (t-test) <0.001

| **A B** |
| --- |
| 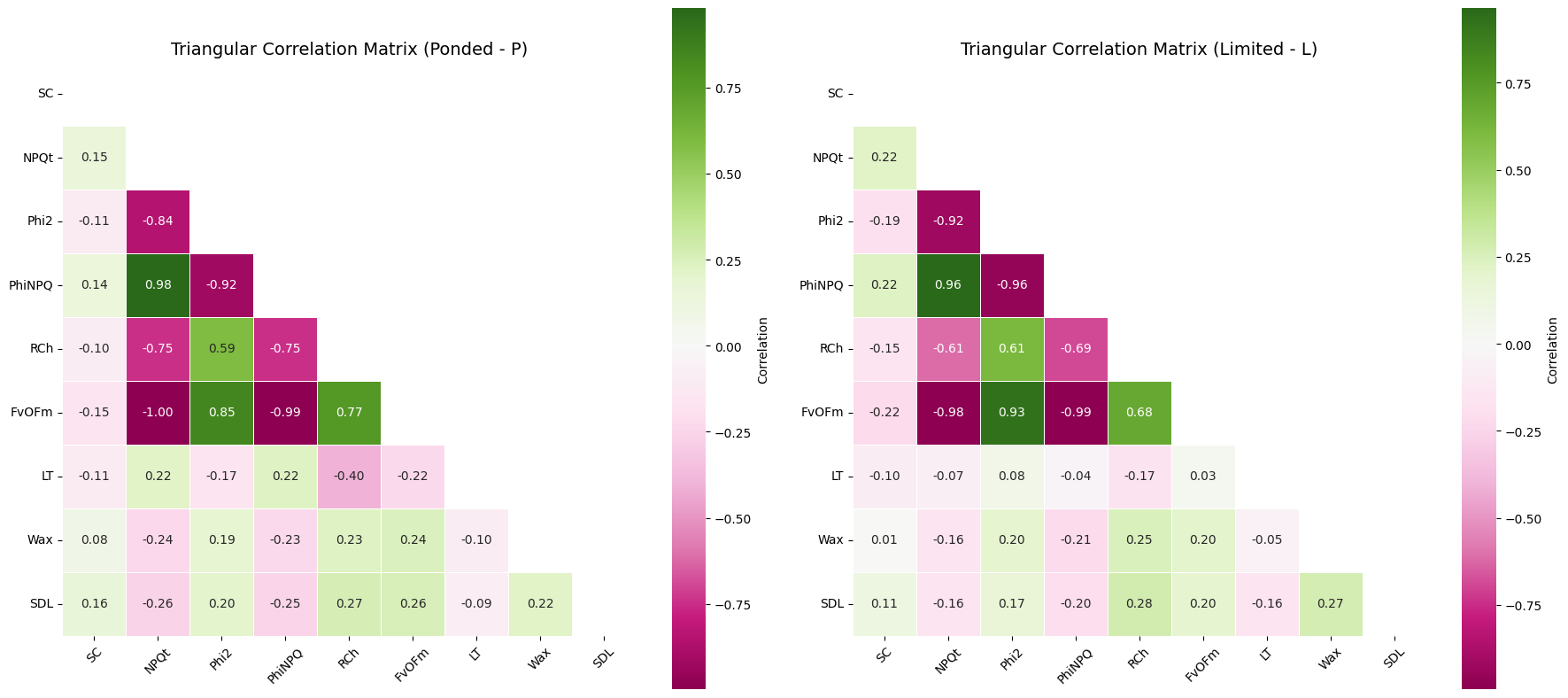 |

**Supplementary Figure 9**. Triangular correlation matrices of rice physiological and structural traits under P (A) and L (B) water treatments (green increasing positive correlation; purple increasing negative correlation).


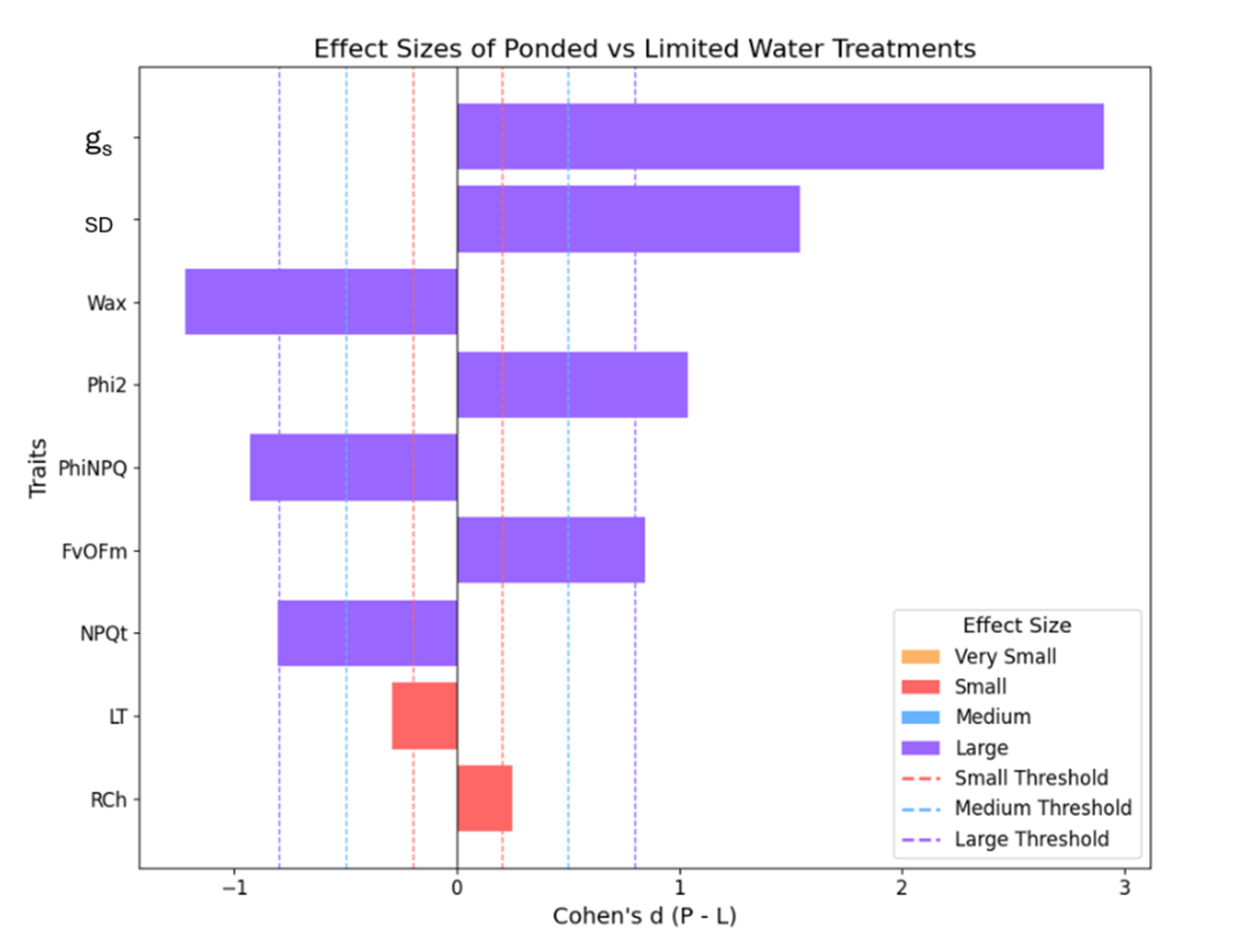


**Supplementary Figure 10**. Cohen’s d effect sizes comparing ponded (PW) and limited water (LW) treatments for measured physiological and structural traits.


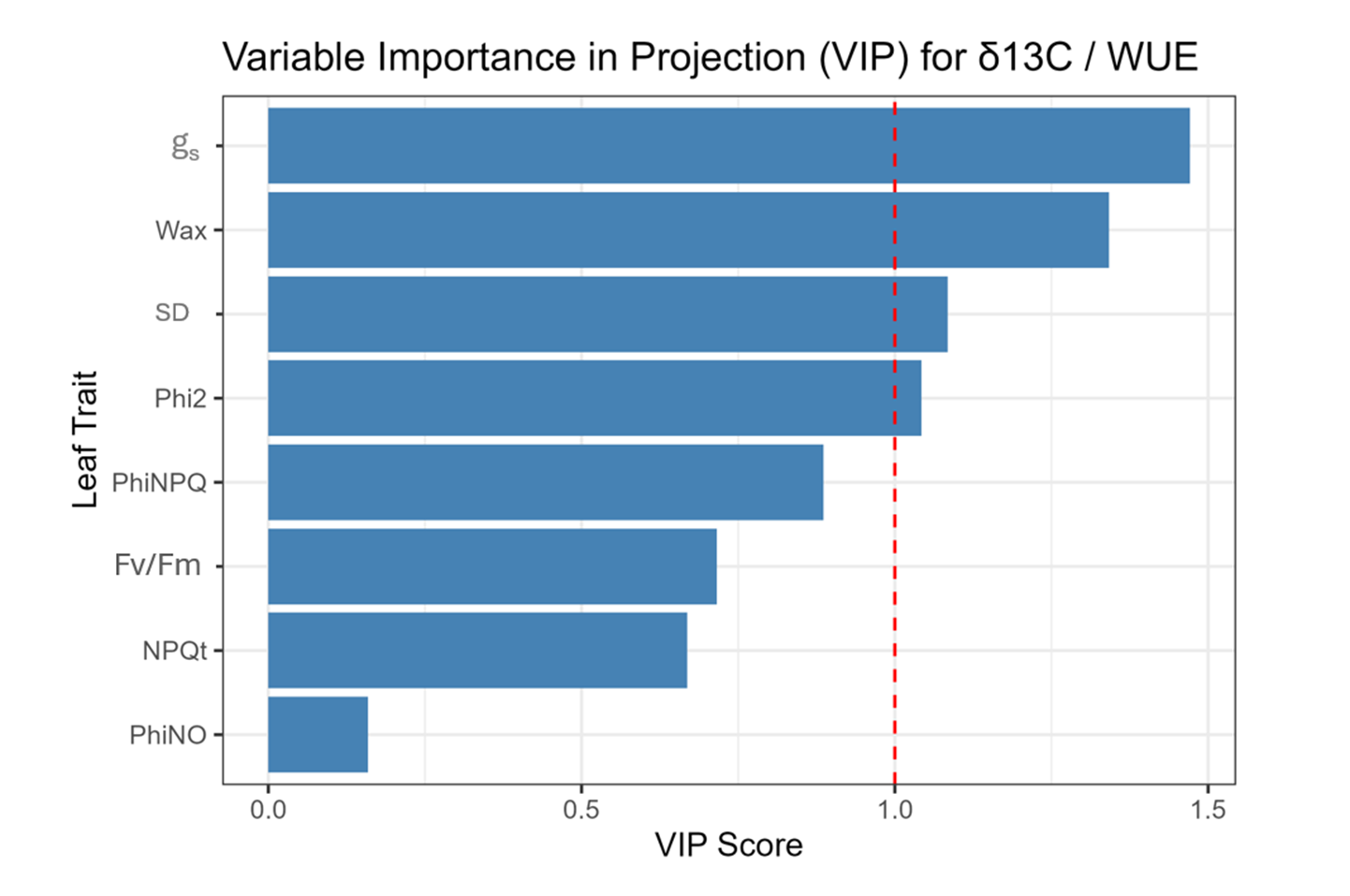


**Supplementary Figure 11**. Variable Importance in Projection (VIP) scores from Partial Least Squares (PLS) regression using δ¹³C as the response variable. Traits with VIP ≥ 1 are identified as strong contributors explaining variation in δ¹³C, while traits with VIP < 1 are considered weak predictors.


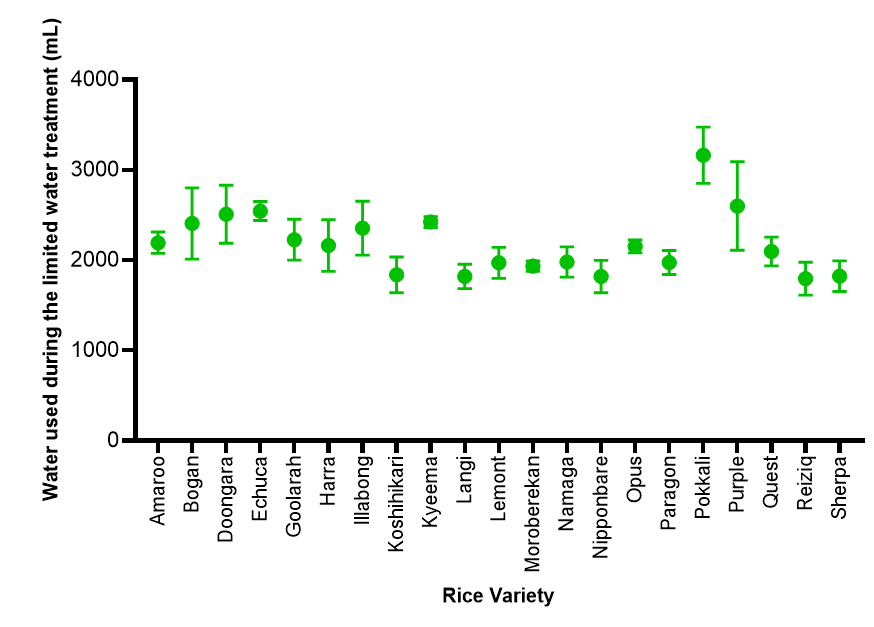


**Supplementary Figure 12.** Whole-plant water use under limited-water (LW) conditions. Genotypes differed significantly in cumulative irrigation requirement, reflecting biological variation in canopy water use and physiological regulation under LW. Indica varieties generally required greater water inputs, consistent with higher tiller number, whereas Sherpa exhibited more conservative water use. These patterns align with leaf trait behaviour and δ¹³C responses, supporting genuine variation in intrinsic WUE. Data represent mean ± SEM, n = 21 varieties with 8 biological replicates.
